# Evidence for ice-free summers in the late Miocene central Arctic Ocean

**DOI:** 10.1038/ncomms11148

**Published:** 2016-04-04

**Authors:** Ruediger Stein, Kirsten Fahl, Michael Schreck, Gregor Knorr, Frank Niessen, Matthias Forwick, Catalina Gebhardt, Laura Jensen, Michael Kaminski, Achim Kopf, Jens Matthiessen, Wilfried Jokat, Gerrit Lohmann

**Affiliations:** 1 Alfred Wegener Institute (AWI) Helmholtz Centre for Polar and Marine Research, Am Alten Hafen 26, Bremerhaven 27568, Germany; 2 Department of Geosciences (FB5), University of Bremen, Klagenfurter Strasse 4, Bremen 28359, Germany; 3 Arctic Research Centre, Korea Polar Research Institute, 26 Songdomirae-ro, Yeonsu-gu, Incheon 406-840, Korea; 4 Institute of Geology, University of Tromsø—The Arctic University of Norway, P O Box 6050 Langnes, Tromsø 9037, Norway; 5 Geosciences Department, College of Petroleum Engineering & Geosciences, King Fahd University of Petroleum & Minerals, Dhahran 31261, Saudi Arabia; 6 MARUM-Center for Marine Environmental Sciences, University of Bremen, Leobener Strasse, Bremen 28359, Germany

## Abstract

Although the permanently to seasonally ice-covered Arctic Ocean is a unique and sensitive component in the Earth's climate system, the knowledge of its long-term climate history remains very limited due to the restricted number of pre-Quaternary sedimentary records. During *Polarstern* Expedition PS87/2014, we discovered multiple submarine landslides along Lomonosov Ridge. Removal of younger sediments from steep headwalls has led to exhumation of Miocene sediments close to the seafloor. Here we document the presence of IP_25_ as a proxy for spring sea-ice cover and alkenone-based summer sea-surface temperatures >4 °C that support a seasonal sea-ice cover with an ice-free summer season being predominant during the late Miocene in the central Arctic Ocean. A comparison of our proxy data with Miocene climate simulations seems to favour either relatively high late Miocene atmospheric CO_2_ concentrations and/or a weak sensitivity of the model to simulate the magnitude of high-latitude warming in a warmer than modern climate.

## Abstract (web summary)

Despite the importance of Arctic sea-ice in the global climate system, a paucity of pre-Quaternary sedimentary archives limits our understanding of its long-term history. Here, based on ancient sediments revealed by submarine landslides, the authors reconstruct Arctic sea-ice conditions during the Miocene.

## Graphical abstract

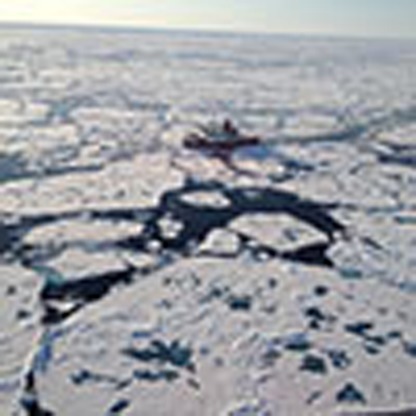

There is a general consensus that the polar regions—and in particular the Arctic Ocean and surrounding areas—are at present, and were over historic and geologic time scales, subject to rapid and dramatic environmental changes. Owing to complex feedback processes, collectively known as ‘polar amplification', the Arctic is both a contributor to climate change and a region that will be most affected by global warming^1^,^2^,^3^. Despite the importance of the Arctic Ocean in the global climate system, this permanently to seasonally ice-covered region (Fig. 1) is one of the last major physiographic provinces on Earth, whose climate history and its transition from early Cenozoic Greenhouse to late Cenozoic Icehouse conditions remain still poorly known. Only one drill site recovered from the central Arctic Ocean during the Arctic Coring Expedition (ACEX)—the Integrated Ocean Drilling Program (IODP) Expedition 302 in 2004—gives some insight into the early Cenozoic climate^4^,^5^,^6^.

Concerning recent climate change, the most prominent example is the dramatic decrease of the extent and thickness of the Arctic sea-ice cover the last decades, a decrease that seems to be by far more rapid than predicted by climate models^1^,^3^. The scientific community recognized this drastic change with major concern as the Arctic sea ice is a critical component in the global climate system, which contributes to changes in the Earth's albedo, primary productivity and deep-water formation, a driving mechanism for global thermohaline circulation^7^. The causes of these recent changes, that is, natural versus anthropogenic forcings, and their relevance within the global climate system, however, are subject of intense scientific and societal debate. Thus, understanding the processes controlling Arctic sea-ice variability is of overall interest and significance^8^,^9^. In this context, records of past climate and sea-ice conditions going beyond instrumental records and representing times of different boundary conditions are of major value: such records can be used to assess the sensitivity of the Earth‘s climate system to changes of different forcing parameters, for example, level of CO_2_, and to test the reliability of climate models by evaluating their simulations for conditions very different from the modern climate. This type of records giving detailed information about past Arctic sea-ice conditions are still very rare, especially due to the lack of precise proxies for sea-ice reconstructions (see review in ref. 9).

The ability to (semi-)quantitatively reconstruct paleo-sea-ice distributions has been significantly improved by a biomarker approach based on the determination of a highly branched isoprenoid (HBI) with 25 carbons (C_25_ HBI monoene=IP_25_)^10^. This biomarker is only biosynthesized by specific diatoms living in the Arctic ice^11^ and appears to be a specific, sensitive and stable proxy for Arctic sea ice in sedimentary sections representing Pliocene to Pleistocene times^12^,^13^. When using this proxy, one has to consider that IP_25_ is absent under a permanent sea-ice cover limiting light penetration and, in consequence, sea-ice algal growth (that is, IP_25_=0). The same consequence applies to totally ice-free conditions (Fig. 1). Müller *et al*.^14^,^15^ overcame this difficulty in interpretating IP_25_ data by the additional use of phytoplankton-derived open-water biomarkers such as brassicasterol or dinosterol (Fig. 1). Furthermore, Müller *et al*.^14^ combined the environmental information carried by IP_25_ and phytoplankton biomarkers in a phytoplankton-IP_25_ index, the so-called ‘PIP_25_ index' (for calculation of PIP_25_, see ‘Methods'). In general, the PIP_25_ values determined in Arctic Ocean surface sediments correlate reasonably well with the modern sea-ice distribution based on satellite data, although regional differences in the correlation patterns seem to be obvious^14^,^16^. In the modern central Arctic Ocean characterized by a mostly perennial sea-ice cover, the PIP_25_ values of most of the surface sediments are between 0.65 and 1 (ref. 16). These data suggest that the PIP_25_ index seems to be a promising proxy for more semi-quantitative estimates of the present and past sea-ice extent, although certainly further groundtruth data are needed (see Methods for some more details)^8^,^16^.

Here we apply the new sea-ice biomarker approach together with alkenone-based sea-surface temperatures (SSTs) to sediment cores most recently recovered during *Polarstern* Expedition PS87 (ref. 17), to reconstruct upper Miocene Arctic Ocean sea-ice and SST conditions. The proxy data are combined with climate model simulations using a coupled atmosphere-ocean general circulation model (AOGCM; see Methods) with focus on seasonal changes in the high northern latitudes. Based on our new proxy records, we demonstrate that only a seasonal sea-ice cover has been predominant in the central Arctic Ocean during (most of) the Late Miocene time interval. Furthermore, our combined data/modelling approach seems to indicate either relatively high atmospheric CO_2_ concentrations and/or an overly weak sensitivity of the model to simulate the magnitude of warm polar temperatures in the late Miocene. These new findings from the Arctic region provide new benchmarks for groundtruthing global climate reconstructions and modelling.

## Results

### Large submarine slide scars along Lomonosov Ridge

*Polarstern* Expedition PS87 was scheduled for August–September 2014 to explore the Lomonosov Ridge area (Fig. 2) with the objective of collecting seismic data and sediment cores to reconstruct the short- and long-term climate history and the tectonic evolution of the central Arctic Ocean^17^. More than 3,000 km of high-quality multi-channel seismic (MCS) profiles and ∼10,000 km of high-quality multibeam bathymetry and sub-bottom sediment-echosounding (PARASOUND) profiles were acquired along the ship's track and numerous sediment cores were recovered (Fig. 2). One major finding of the expedition was the discovery of numerous submarine slide scars that occurred on both sides of the crest of Lomonosov Ridge over a distance of *ca*. 350 km between 81°07' N and 84°14' N in water depths from ∼800 to 1,500 m (Fig. 3a). Single scars are up to several kilometres wide and long, and their head walls are 100–500 m high. Swath-bathymetry data indicate that different processes probably triggered slope failures, that various processes of sediment evacuation took place, and that failures occurred at various times. Slide scars were earlier described from a restricted area on Lomonosov Ridge near 88°N^18^. However, the wide lateral distribution of mass wasting as presented here is a new discovery.

On top of the southern Lomonosov Ridge in areas between the slide scars, we discovered SE–NW oriented, streamlined landforms over distances of >100 km at water depths between 800 and 1,000 m (Fig. 3a). These features are interpreted to be glacial lineations that formed beneath grounded ice^19^,^20^. Similar unidirectional bed forms have also been identified further east on the East Siberian continental margin where they were related to large and coherent ice masses (East Siberian Chukchi Ice Sheet—ESCIS)^21^. The lineations identified in this study are similar to those on the East Siberian continental margin with respect to their orthogonal orientations to the proposed centre of the former ESCIS. At the present state of knowledge, the most plausible glacial scenario is a larger than originally proposed ESCIS including an ice shelf extending into the Arctic Ocean, which formed an ice rise on the Southern Lomonosov Ridge over areas presently shallower than 1,000 m. The load and erosional behaviour of this ice rise that probably occurred during extended Quaternary glaciations, for example, during Marine Isotope Stage 6 (MIS 6), may have caused physical conditions that triggered the landslides on this part of Lomonosov Ridge. A MIS 6 age of this erosional event is in line with the proposed age of a major glaciation with extended ice sheets/shelves in Eurasia and East Siberia^19^,^20^,^21^.

### Exhumation of Miocene to lower Quaternary sediments

Sediment removal from the steep slopes of the escarpments exposed older, normally more deeply buried deposits at/near the present seafloor, allowing retrieval of older sediments by gravity coring from *Polarstern*, rather than expensive drilling. The PARASOUND and MCS profiles suggest that these sedimentary sections are composed of Eocene, Oligocene, Miocene, Pliocene and Quaternary strata (Fig. 3b,c). The age control for the stratigraphic units is based on links of seismic lines to drill site data from the Chukchi Shelf, the ACEX drill site on central Lomonosov Ridge and onshore geology from the New Siberian Islands^22^,^23^. After evaluation of the multibeam bathymetry and PARASOUND data, we selected two transects across the steep western slope of Lomonosov Ridge for an extensive sediment coring programme (Fig. 3a, Transect 1 and Transect 2). In total, 16 sediment cores were recovered from water depths between 900 m (top of Lomonosov Ridge) and 1,500 m (foot of Lomonosov Ridge)^17^ (Fig. 3a,b and Supplementary Table 1).

**Figure 1:**
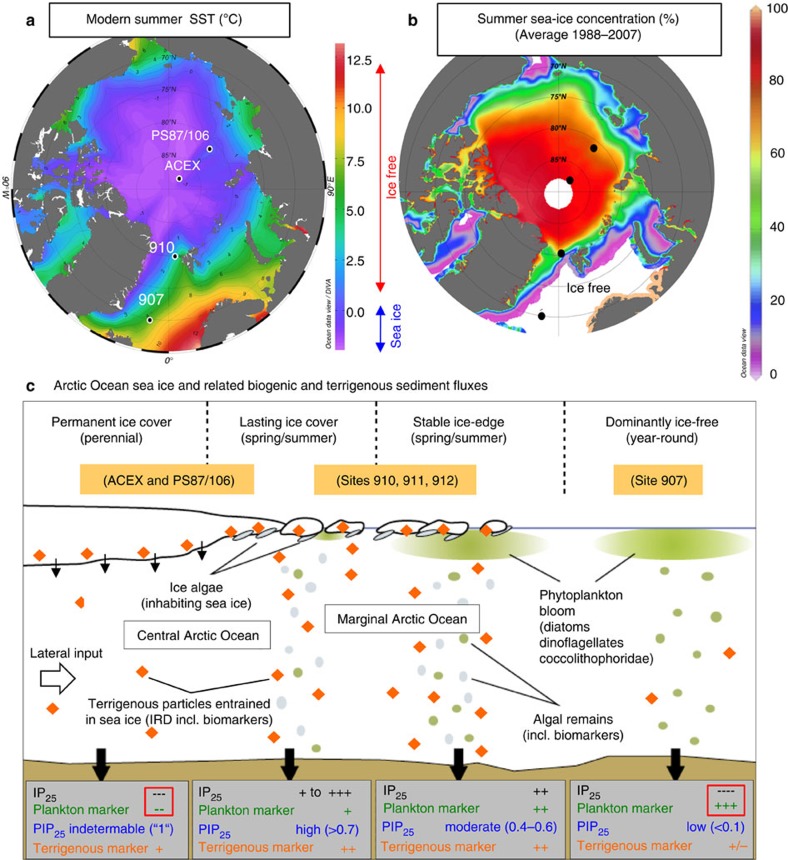
Modern Arctic Ocean SST and sea-ice conditions. (**a**) Map of modern August SST with locations of ODP Site 907, ODP Site 910 and IODP Expedition 302 (ACEX) Site, as well as Core PS87/106 (August SST data—average of 1955–2012—from World Ocean Atlas; http://odv.awi.de/en/data/ocean/world_ocean_atlas_2013/). (**b**) Map of summer sea-ice concentration—average of 1988–2007—with locations of studied sites (black dots); data source: http://nsidc.org. Maps **a** and **b** produced with Ocean Data View software (source: http://odv.awi.de/). (**c**) Generalized scheme illustrating (1) sea surface conditions and respective (spring/summer) productivities of ice algae and phytoplankton, and (2) sedimentary contents of IP_25_, terrigenous biomarkers and IRD, phytoplankton-derived biomarkers and PIP_25_ index for different settings in the modern Arctic Ocean (after ref. 14, supplemented). Owing to ice melting and related nutrient and sediment release, a stable ice-edge situation is characterized by high concentrations of IP_25_ and phytoplanktion biomarkers, but also by high concentrations of terrigenous biomarkers and IRD. Modern situations at locations of ACEX and PS87/106, ODP Site 910 (and close-by ODP sites 911 and 912) and ODP Site 907 are indicated.

Whereas most of the sediment cores of Transect 1 are composed of predominantly upper Quaternary (post slide) deposits, some contain prominent unconformities with lower/pre-Quaternary sediments underneath (see Methods and Supplementary Figs 1 and 2). At these unconformities, a 50- to 80-m-thick overburden has been removed, as demonstrated in compaction experiments (see ‘Sediment load and compaction experiments'). Unfortunately, the microfossil assemblages (that is, palynomorphs and agglutinated benthic foraminifers) do not allow a precise age determination of the sediments underlying the Quaternary near-surface deposits in most of these cores (Supplementary Table 1). The predominance of Quaternary sediments in the cores of Transect 1, however, seems to be supported by the biomarker composition determined in selected cores. Close to zero concentrations or the absence of specific biomarkers indicative for phytoplankton and sea-ice algae productivity point to surface-water conditions similar to those of the central Arctic Ocean during late Quaternary times^16^, characterized by a thick perennial sea-ice cover and SSTs <<0 °C (Supplementary Table 2 and Fig. 1).

The only core providing a clear indication that old sediments are cropping out near the seafloor is Core PS87/106. In this core recovered at the lower slope at Transect 2 (Fig. 3a), a sharp unconformity or hiatus occurs at 370 cmbsf. This unconformity is characterized by a marked change in colour, an abrupt increase in wet-bulk density (WBD; related to enhanced sediment consolidation underneath the hiatus), a significant drop in magnetic susceptibility (MS) and a major change in the biomarker composition, and it separates this interval from the overlying young and unconsolidated (upper Quaternary) sediments (Fig. 4 and Supplementary Table 2). The common occurrence of the acritarch *Decahedrella martinheadii* clearly indicates a late Miocene age for the lower part of the sedimentary sequence (Supplementary Fig. 3). The co-occurrence of the dinoflagellate cyst *Nematosphaeropsis labyrinthus* in combination with the absence of the *Batiacasphaera micropapillata* complex, however, may suggest a placement of the PS87/106 sediments within the upper part of its stratigraphic range, that is, upper Tortonian to lower Messinian, when compared with IODP/ACEX Hole M2A and ODP Hole 907A^24^,^25^,^26^,^27^. Reworking of these late Miocene palynomorphs is excluded due to excellent preservation of the encountered delicate palynomorph specimens (see Methods for details of the age model of Core PS87/106).

### Late Miocene SST and sea-ice records

The biomarker data of Core PS87/106 suggest significantly different late Miocene paleoenvironmental conditions in comparison with those predominant during Quaternary times (Fig. 4 and Supplementary Table 2). In the upper Miocene sediments, elevated concentrations of alkenones and alkenone-derived SST between 4 and 7 °C (or even 6–9 °C in case other calibrations are used; *cf*. Supplementary Table 2) indicate relatively warm, open-water and productive paleoenvironmental conditions in the central Arctic Ocean during the summer season. This is also supported by SST values of »0 °C determined in some samples from the ACEX Site (Supplementary Tables 2 and 3).

Our results reveal for the first time the occurrence of the biomarker sea-ice proxy IP_25_ in sediments as old as late Miocene. This proxy was developed by Belt *et al*.^10^ and was before our study only found in Quaternary and Pliocene sediments^12^,^13^. The presence of IP_25_ in the PS87/106 sediments is indicative for the presence of (spring) sea ice in the late Miocene central Arctic Ocean (Fig. 1). In comparison with IP_25_ values from the Arctic Ocean surface sediments^16^ and sediment trap data^28^, the absolute IP_25_ concentrations ranging between 0.05 and 0.15 μg gOC^−1^ (Fig. 4 and Supplementary Table 2) are more than one order of magnitude lower. These differences are caused by an early degradation of biomarkers that already starts in the water column and reaches its maximum in the uppermost centimetres of the sediments^28^,^29^. On the other hand, both IP_25_ and phytoplankton biomarker concentrations determined in Core PS87/106 are in the same range than those determined in early-mid Holocene Arctic sediments^8^,^30^.

Using the ‘PIP_25_ Index' as a more semi-quantitative proxy of paleo-sea-ice cover (see Fig. 1 and Methods), our data from Core PS87/106 point to a variable spring sea-ice coverage of ∼20–70% in the lower part and ∼100% in the upper part of the sequence (Fig. 4). The combination of IP_25_ and SST data indicates that the central Arctic Ocean must have been relatively warm and ice-free during summer throughout the time interval recovered in the sedimentary section of Core PS87/106 and variable sea ice must have existed during spring when daylight conditions allowed sea-ice algae production (Figs 1 and 5, and Supplementary Table 2). Furthermore, this implies the presence of an extended sea-ice cover during the dark, cold winter season. These new data clearly support that periods with only a seasonal sea-ice coverage must have occurred in the central Arctic Ocean during most of the late Miocene (Fig. 5; see further discussion below).

### Simulations of late Miocene Arctic Ocean climate

Model simulations of global climate conditions reconstructing a warm late Miocene climate apply atmospheric CO_2_ concentrations in the range of 280–700 p.p.m.^31^,^32^,^33^,^34^. Different reconstructions of late Miocene CO_2_ levels have narrowed the uncertainties, with atmospheric CO_2_ values likely to be below the present day concentrations^35^,^36^. Nevertheless, the associated uncertainties in the late Miocene CO_2_ levels are at least 200 p.p.m.^34^,^37^,^38^. Therefore, we have re-analysed Miocene climate simulations^32^,^39^ with CO_2_ concentrations of 278 and 450 p.p.m. using a coupled AOGCM. The investigation focus is on seasonal changes in the central Arctic Ocean as simulated with the AOGCM^32^,^39^ (for details and background, see Methods).

**Figure 2:**
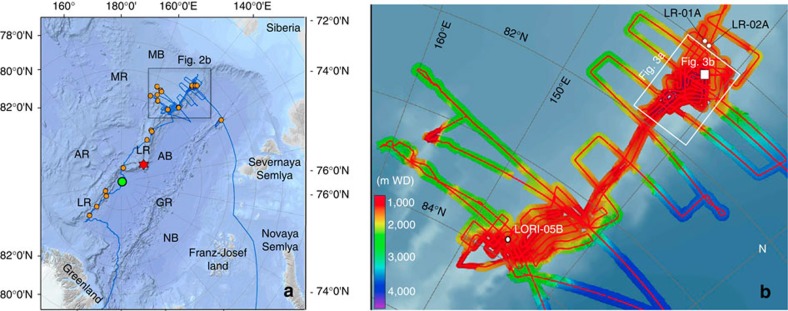
Cruise track and multibeam bathymetric survey of *Polarstern* Expedition PS87. (**a**) PS87 cruise track (blue line)^17^. AB, Amundsen Basin; AR, Alpha Ridge; GR, Gakkel Ridge; LR, Lomonosov Ridge; MB, Makarov Basin; MR, Mendeleev Ridge; NB, Nansen Basin. Orange circles indicate coring stations, the red asterisk indicates the location of the ACEX Site and the green circle indicates the North Pole (*Polarstern* reached on 26 August 2014 at 10:23 UTC). (**b**) Track lines of multibeam bathymetric survey. Colour bar indicates water depth in metres (m WD). LR-01A, LR-02A and LORI-05B indicate locations of proposed IODP drill sites^51^ (*cf*., IODP Proposal 708; http://www.iodp.org/expeditions). Areas of Fig. 3a,b are indicated.

Our simulated mean August SST and mean sea-ice concentrations for March, June and September/August for high (450 p.p.m.) and low (278 p.p.m.) CO_2_ levels (Fig. 6 and Supplementary Table 4) indicate the following: (1) a winter season with a closed sea-ice cover in the central Arctic Ocean decreasing towards the marginal seas for 450 p.p.m. CO_2_ (Fig. 6a) and a closed sea-ice cover in the entire Arctic Ocean including the marginal seas for 278 p.p.m. CO_2_ (Fig. 6d); (2) spring season sea-ice concentrations of 20–60% for 450 p.p.m. CO_2_ (Fig. 6b) and even 80–90% for 278 p.p.m. CO_2_ (Fig. 6e); and (3) a summer season with ice-free conditions and SSTs >0 °C for 450 p.p.m. CO_2_ (Fig. 6c,g) and a reduced but still present sea-ice cover of 10–50% and SSTs <−1 °C for 278 p.p.m. CO_2_ (Fig. 6f,h).

The AOGCM-based monthly mean sea-ice concentrations for 450 and 278 p.p.m. CO_2_ levels for late Miocene and preindustrial conditions at the ACEX, PS87/106, ODP 910 and ODP 907 sites are shown in Fig. 7 and—together with SST values—listed in Supplementary Table 4. Based on these results, the central Arctic Ocean was more or less ice free during summer and autumn (that is, between mid-June to mid-November) for 450 p.p.m. CO_2_, whereas sea ice still existed during summer times for 278 p.p.m. CO_2_ simulations. At the location of ODP Site 907, ice-free conditions occurred throughout the year for both simulations. In addition to the late Miocene and preindustrial simulations, future monthly mean sea-ice concentrations for AD 2100 and AD 2200 (following the Intergovernmental Panel on Climate Change medium stabilization scenario RCP4.5) (ref. 40) are shown (Fig. 7). These simulations suggest that the summer sea-ice concentrations will become significantly reduced to 20–40% during the next two centuries.

In combination with our new SST and sea-ice proxy data, these results suggest that either late Miocene CO_2_ levels have been relatively high or, alternatively, the applied model has an overly weak sensitivity especially in the northern high latitudes, as in the simulation with a relatively low CO_2_ level of 278 p.p.m. summer SSTs are too low to explain our sedimentary SST proxy indicative of 4–7 °C. (Fig. 4 and Supplementary Tables 3 and 4). The latter reflects a characteristic challenge of current model approaches to simulate warm climates in the geological past^41^,^42^. Hence, the new findings from the Arctic region provide an enhanced basis for groundtruthing global climate reconstruction and modelling.

## Discussion

Late Miocene climatic conditions significantly warmer than today have been reconstructed from marine and terrestrial proxy records from different localities around the globe^43^,^44^,^45^,^46^,^47^. However, quantitative SST proxy data from the High Arctic are exclusively restricted to a few terrestrial records^48^,^49^. With our study, we show for the first time that the late Miocene central Arctic Ocean was relatively warm with SSTs of ∼5 °C (Figs 4 and 8) and ice-free during summer, whereas sea ice occurred during spring and autumn/winter. During the late Miocene a general cooling trend is obvious as recorded in the SST record of the sub-Arctic ODP Site 907 (Fig. 8 and Supplementary Table 5). Such a cooling trend seems to be supported by the limited number of SST values available from the ACEX site (Fig. 8). Comparing this general cooling trend with the SST values from Core PS87/106, the sedimentary section of Core PS87/106 below the hiatus probably represents a time span within the upper Tortonian to lower Messinian as supported by palynomorph stratigraphy (Fig. 8). Furthermore, the new Arctic SST data fit in very well with the grander long-term Cenozoic cooling pattern (see Supplementary Fig. 4 and references in the figure legend).

Although on a first view this short sedimentary section of Core PS87/106 only represents a short snapshot of late Miocene Arctic climate, more detailed information about the late Miocene climate on a regional to even global scale can be obtained from our record. Based on the biomarker data, the 1.3-m-thick late Miocene section of Core PS87/106 probably represents almost one cycle with extended and reduced spring sea-ice conditions (Fig. 4). The (almost) absence of phytoplankton biomarkers and IP_25_ (PIP_25_= ‘1'; see Fig. 1) and low concentrations of terrigenous biomarkers may be explained by an extended to closed sea-ice cover and a very restricted spring season (Scenario 1 in Figs 4 and 5). Scenario 2, on the other hand, represents a transitional phase with a stable ice edge during an extended and productive spring season, characterized by maximum input of phytoplanktion biomarkers and IP_25_ (resulting in PIP_25_ values of 0.4–0.7), as well as maximum input of terrigenous biomarkers (and ice-rafted debris (IRD)) (Scenario 2 in Figs 4 and 5). The interval between scenarios 1 and 2 is characterized by very low to zero IP_25_ concentrations and increased concentrations of phytoplankton biomarkers, resulting in a distinct PIP_25_ minimum (Fig. 4). This interval is interpreted as a period of minimum spring sea-ice extent. Furthermore, maximum values of alkenones may reflect increased productivity of haptophyte algae during the summer season.

Using mean sedimentation rates of ∼3.2 cm ky^−1^ as calculated independently from close-by gravity cores^50^ and seismic data^51^, the duration of this cycle is about 40 ky, that is, very similar to the 41 ky obliquity cycle (Fig. 4 and also see ref. 52 and references therein). Hence, our record may represent just one obliquity cycle with ice-free conditions during summers in both the cold (‘glacial') and the warm (‘interglacial') phase of this climate cycle (Figs 4 and 5). As Core PS87/106 probably is of upper Tortonian to lower Messinian age (see above), ice-free summer conditions should have occurred in the central Arctic Ocean during the warmer Middle Miocene to early Late Miocene time interval *a fortiori* (Fig. 8). In contrast to several previous studies, we therefore propose that a seasonal sea-ice cover was predominant in the central Arctic Ocean during (most of) the late Miocene time interval (see discussion below).

Our new semi-quantitative sea-ice and SST records are an important contribution to the ongoing and controversial debate about the reconstruction of the early (pre-Quaternary) Arctic Ocean sea-ice cover. Within this debate, the distinction between seasonal and perennial sea ice is critical, because year-round sea ice in the central Arctic implies very different climate feedback mechanisms, that is, Earth's albedo and heat exchange conditions, than an environment with ice-free conditions during summer. As outlined above, our new proxy data and modelling data clearly indicate a late Miocene seasonal sea-ice cover. Similar or even warmer climatic conditions also occurred in the Middle Miocene Arctic Ocean when looking at the alkenone-based summer SSTs of 10–13 °C reconstructed from ACEX sediments (Supplementary Fig. 4 and also see ref. 53). Abundant marine palynomorphs and foraminifers found in Miocene and early Pleistocene sediments of the ACEX section also point to at least periods with seasonally ice-free conditions^24^,^54^. These data are in contrast to an Arctic Ocean perennial sea-ice cover from middle Miocene onwards, as proposed by Darby^55^ and Krylov *et al*.^56^ based on their provenance studies of IRD in ACEX sediments. Taking the IRD with a North American or East Siberian origin found in the ACEX sediments and using modern sea-ice drift trajectories and velocities, these authors concluded that more than 1 year was needed to transport the sediments entrained in the sea ice to the ACEX location. Hence, the sea ice must have survived the summer melting season to reach the ACEX site.

Using a ‘coupled sea-ice slab-ocean model including sediment transport by sea ice', Tremblay *et al*.^57^ have most recently demonstrated that sea-ice drift was probably significantly faster under warmer climatic conditions with less or much thinner sea ice than today. They conclude that the presence of ice-rafted sediment of Eurasian and North American origin at the North Pole is not a definite indication of a perennial sea ice cover in the Arctic Ocean. This may demonstrate that the main assumption of Darby^55^ and Krylov *et al*.^56^ should be regarded critically.

Our proxy and model reconstruction of late Miocene Arctic climate is certainly a substantial step forward to improve the understanding of the pre-Quaternary Arctic Ocean sea-ice and SST history. However, to decipher the pre-Quaternary climate history of this unique and sensitive but still not well-known region on Earth in more detail, long continuous sedimentary records to be obtained only by scientific drilling are needed. These records are planned to be recovered within a new IODP drilling campaign scheduled for 2018 (IODP Proposal 708; http://www.iodp.org/expeditions). Based on the new PS87 seismic data, ∼200 m of Plio–Pleistocene, >600 m of Miocene and >300 m of Oligocene–Eocene may be recovered at the proposed drill sites^51^ (see Fig. 2 for locations). The outcome of such a new programme will certainly help to improve our understanding of the complex ocean–atmosphere–ice system in the polar high northern latitudes and its role in the past, modern and future global climate.

## Methods

### Shipboard surveys and measurements

The methods used on board *Polarstern* during Expedition PS87 are shortly outlined in the following. For a more detailed information about the use and interpretation of the proxy data we refer to the different chapters of the Cruise Report^17^.

The bathymetric survey was performed using the hull-mounted ATLAS Hydrographic HYDROSWEEP DS3, a deep-sea multi-beam swath sonar system with a resolution of up to 320 receive beams per ping, a swath width of 4–5 times the water depth and a vertical resolution of ∼0.5% of the water depth. It was operated in the chirp mode with a frequency of 14–16 kHz. The mean sound velocity of the water column was calculated from conductivity-temperature-density (CTD), expandable CTD (XCTD) and Valeport Sound Velocity Profiler data.

**Figure 3:**
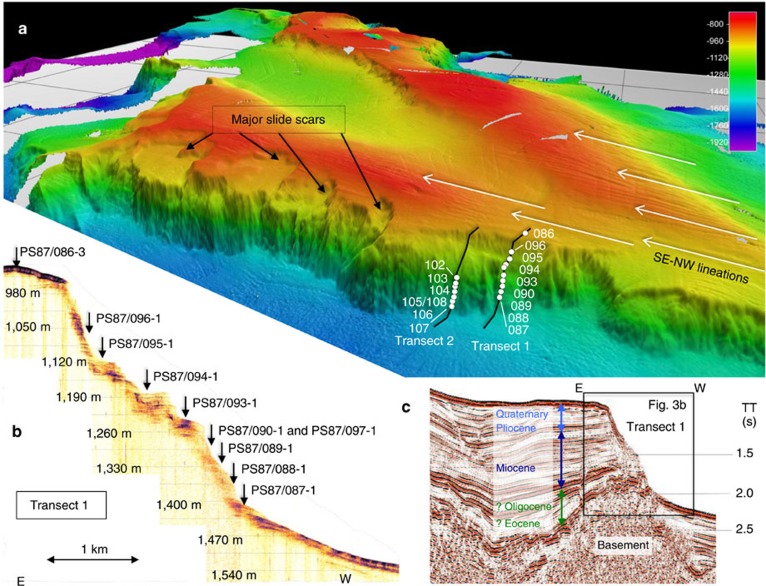
Bathymetric and acoustic/seismic profiling records from southern Lomonosov Ridge. (**a**) Three-dimensional (3D) image of the swath bathymetry of southern Lomonosov Ridge, showing major slide scars and escarpments, streamlined SE–NW oriented glacial lineations formed beneath grounded ice sheets/streams and transects 1 and 2 with locations of sediment cores (*cf*., Supplementary Table 1). (**b**) PARASOUND profile across Transect 1 with locations of coring stations. (**c**) Processed multi-channel seismic profile AWI-20140311 across Transect 1, showing prominent reflectors and age assigments based on correlation between regional seismic lines and well data^22^,^23^.

Sub-bottom profiling data were acquired using the parametric hull-mounted system ATLAS Hydrographic PARASOUND DS III-P70. Primary operating frequencies were 18.75 and 22.95 kHz with a secondary sediment-penetrating frequency of 4.2 kHz, a beam angle of 4° and a pulse length of 2. The vertical resolution is ∼0.2 m. As a result of the narrow beam angle, reflections from strata dipping by >4° cannot be received by the vessel. This explains why the thin veneer of post-slide sediments covering the slump scar is not resolved as it is above and below the headwall (Fig. 3b). The headwall has an inclination >4°. In contrast, the older (pre-Quaternary) sediments exhibit nearly horizontal bedding (Fig. 3), which are thus acoustically resolved along the headwall to their near-seafloor location. PARASOUND data visualization and processing was performed using ATLAS PARASTORE-3 software. The vertical scale on profiles has been converted from travel time to metres using a constant sound velocity of 1.5 km s^−1^, which explains minor differences in water depth between PARASOUND and swath-sonar data.

For the MCS data acquisition, a 3,000-m-long streamer (240 active channels, group interval of 12.5 m) and an air gun array of four G-Guns (total volume of 32 l, fired with 200 bar every 15 s) were used. Processing included sorting, that is, common depth point sorting with 25 m spacing, frequency filtering (20–180 Hz), velocity analysis, multiple suppression and stacking.

Whole-core measurements included non-destructive, continuous determinations of core geometry (diameter), WBD, P-wave velocity (*V*p) and loop-sensor MS at 10 mm intervals, using a standard Multi-Sensor Core Logger (GEOTEK Ltd., UK). The principle of logging cores is described in more detail in the GEOTEK manual ‘Multi-Sensor Core Logging', which can be downloaded from the web (http://www.geotek.co.uk).

Line-scan images (Supplementary Figs 1 and 2) were acquired with a Jai CV L107 camera with RGB (red-green-blue) channels at 630, 535 and 450 nm, respectively, mounted to an Avaatech XRF core scanner. The camera contains three charge-coupled device sensors and a beam splitter to separate the RGB signal. Images were acquired with a down-core resolution of ∼70 μm.

### Stratigraphic framework and marine palynology process

The general lithostratigraphic framework and age model of the upper Quaternary sedimentary sections recovered during Expedition PS87 are robust and based on lithostratigraphy, colour imaging, WBD and MS records characterized by very prominent minima and maxima, and correlation with other dated sediment cores from Lomonosov Ridge^17^,^50^. Based on this concept, MIS 6 to 1 were identified in most of the cores (Supplementary Fig. 1). Some cores from very steep sections of the slope along Transect 1 (Fig. 3b), on the other hand, contain multiple unconformities and a correlation to the reference Core PS87/086 was only partly possible (Supplementary Fig. 2). WBD typically increases sharply below the unconformities due to higher degrees of compaction, supporting a hiatus and ‘older' sediments below (Supplementary Fig. 2). For this study dealing with the late Miocene climate history, only the identification and dating of these ‘older' (Neogene) sedimentary sections are relevant. For assessing the age of these sediments, assemblages of agglutinated benthic foraminifers and palynomorphs were used and compared with biostratigraphic records obtained from the ACEX and North Atlantic sites^17^,^24^,^25^,^26^,^27^ (Supplementary Table 1).

For sample preparation and processing of the agglutinated benthic foraminifers we refer to the PS87 Cruise Report^17^ and further references therein. The procedure for studying the palynomorphs are as follows: dinoflagellate cysts and acritarchs were investigated in 25 samples (mainly core catcher) from 15 cores recovered along transects 1 and 2 (Fig. 3a and Supplement Table 1), with special emphasis on Core PS87/106. Sediment was freeze dried, weighed and processed using standard palynological maceration techniques including repeated treatment with cold HCl (10%) and cold HF (38–40%), no oxidation and sieving over a 10-μm-nylon mesh. The residue has been mounted with glycerine jelly on microscope slides, which were then scanned for dinoflagellate cysts and acritarchs using a light microscope at × 400 original magnification.

### Age model of the late Miocene section of core PS87/106

Based on microfossil data (that is, palynomorphs and agglutinated benthic foraminifers), the core catcher samples from the sediment cores were barren or give a Pleistocene age (Supplementary Table 1). The only core providing any clear indication that old sediments are cropping out in the shallow sub-seafloor is Core PS87/106. Well-preserved specimens of organic-walled palynomorphs (dinoflagellate cysts and acritarchs) have been recorded in successive samples from the base of the core (including core catcher) up to 420 cmbsf (that is, 50 cm below the hiatus; Supplementary Table 1). Of the encountered species, the acritarch *D. martinheadii* provides evidence that the lower part of the core is composed of sediments of late Miocene age. This species is endemic to the high northern latitudes and its stratigraphic range has been discussed previously based on comprehensive reviews of its occurrence at several DSDP, ODP and IODP sites from the Central Arctic Ocean, Norwegian-Greenland Sea, Labrador Sea, Baffin Bay and Irminger Sea^24^,^25^,^26^. It is restricted to the late Miocene in the Arctic and subarctic realm, and based on the pristine paleomagnetic record of Iceland Sea ODP Site 907, its stratigraphic range is independently calibrated against the astronomically tuned Neogene Timescale, thus providing absolute age control (Supplementary Fig. 3). A near-synchronous highest occurrence at *ca*. 6.3–6.2 Ma has been defined from several northern high latitude sites, suggesting this species to be an excellent marker across the subpolar/polar North Atlantic and Arctic Ocean^25^. In addition, a highest common occurrence is recognized at 6.5 Ma in Iceland Sea ODP Hole 907A and more generally at *ca*. 6.7–6.3 Ma across the Norwegian-Greenland Sea. The lowest occurrence of *D. martinheadii* has been calibrated to 10.5 Ma in ODP Hole 907. According to Schreck *et al*.^25^, however, the lowest occurrence is not very well constrained at other sites but certainly younger than 11 Ma across the northernmost North Atlantic and Arctic Ocean.

In Core PS87/106, the consistent occurrence of *D. martinheadii* is accompanied by low numbers of the dinoflagellate cyst *N. labyrinthus*. Such co-occurrence has also been observed within the upper part of its stratigraphic range in IODP Hole M2A^24^ and ODP Hole 907 (refs 25, 27). Furthermore, the *B. micropapillata* complex has not been recorded in PS87/106 samples. This dinoflagellate cyst dominates assemblages in the late Serravallian of IODP/ACEX Hole M2A, decreases significantly across the Tortonian and disappears close to the Tortonian/Messinian boundary^26^. In ODP Hole 907A, *B*. *micropapillata* complex dominates the assamblage until *ca*. 8.2 Ma after which it only occurrs sporadically until its highest common occurrence at *ca*. 4.5 Ma^26^,^27^. Therefore, the co-occurrence of *D. martinheadii* and *N. labyrinthus* in combination with the absence of *B. micropapillata* complex may allow to place the analysed interval of Core PS87/106 into the upper Tortonian to lower Messinian. However, we note that the highest occurrence derived from ODP Site 907 may represent a minimum age for this bioevent in the Central Arctic Ocean, as successive Neogene cooling may led to an earlier disappearance of species in the higher latitudes.

*D. martinheadii* and *N. labyrinthus* are both very delicate species that bear processes and trabeculae (ribbon-like bars), which tend to crumple easily. All specimens encountered during palynological analyses, however, are well preserved indicating *in situ* deposition.

### Biomarker analyses

Extraction of 5–10 g of freeze-dried sediments was carried out using an accelerated solvent extractor (DIONEX, ASE200; 100 °C, 5 min, 1,000 psi) with dichloromethane:methanol (2:1, v/v) as the solvent. For quantification internal standards, 7-hexylnonadecane (7-HND, 0.076 μg per sample for IP_25_ quantification), squalane (2.4 μg per sample) and cholesterol-d_6_ (cholest-5-en-3β-ol-D_6_, 10 μg per sample for sterol quantification) were added before analytical treatment. Separation of the hydrocarbon and sterol fractions was carried out via open column chromatography (hydrocarbon fraction with 5 ml *n*-hexane, the sterol fraction with 6 ml *n*-hexane:ethylacetate (5:1, v/v)). The latter fraction was silylated with 500 ml BSTFA (bis-trimethylsilyl-trifluoroacet-amide) (60 °C, 2 h). IP_25_ and sterols were analysed by gas chromatography (GC)/mass spectrometry. Component assignment was based on comparison of GC retention times with those of reference compounds and published mass spectra (Supplementary Figs 5 and 6). The Kovats Index calculated for IP_25_ is 2,086. For the monounsaturated HBI alkene (HBI monoene) most recently found in ancient Arctic sediments and characterized by very similar chromatographic and mass spectral properties^58^, the Kovats Index has been calculated as 2,090. As this new HBI monoene is absent in the investigated cores of this study, we have calculated the index from lower Pliocene sediment samples from ODP Site 911 to show that both compounds can clearly be separated by our analytical approach (see Supplementary Fig. 5a,b). The detection limit for quantification of IP_25_ (Agilent 7890B GC, Agilent 5977A Extractor MSD with Performance Turbo Pump) is 0.005 ng μl^−1^ in SIM (selected ion monitoring) mode. To obtain mass spectra in TIC (total ion current) the limit is 0.05 ng μl^−1^. The retention indices for brassicasterol (as 24-methylcholesta-5,22E-dien-3β-O-Si(CH3)3), campesterol (as 24-methylcholest-5-en-3β-O-Si(CH3)3) and β-sitosterol (as 24-ethylcholest-5-en-3β-O-Si(CH3)3) were calculated to be 1.018, 1.042 and 1.077 (normalized to cholest-5-en-3β-ol-D_6_ set to be 1.000), respectively.

For the quantification of IP_25_, its molecular ion (*m/z* 350) in relation to the abundant fragment ion *m/z* 266 of the internal standard (7-HND) was used (SIM mode). The different responses of these ions were balanced by an external calibration (Supplementary Fig. 5c and also see ref. 28). Brassicasterol (24-methylcholesta-5,22E-dien-3β-O-Si(CH_3_)_3_), campesterol (24-methylcholest-5-en-3β-O-Si(CH_3_)_3_) and β-sitosterol (24-ethylcholest-5-en-3β-O-Si(CH_3_)_3_) were quantified as trimethylsilyl ethers using the molecular ions *m/z* 470, *m/z* 472 and *m/z* 486, respectively, in relation to the molecular ion *m/z* 464 of cholesterol-D_6_.

More details about the identification and quantification of IP_25_ and the sterols are described elsewhere^8^,^28^,^29^,^58^,^59^,^60^,^61^.

For more semi-quantitative estimates of the present and past sea-ice coverage, Müller *et al*.^14^ combined the sea-ice proxy IP_25_ and phytoplankton biomarkers in a phytoplankton-IP_25_ index, the so-called ‘PIP_25_ index' (Fig. 1):


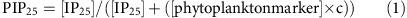


with *c*=mean IP_25_ concentration/mean phytoplankton biomarker concentration for a specific data set or core. As phytoplankton biomarkers brassicasterol and dinosterol were used, resulting in P_bras_IP_25_ and P_dino_IP_25_ values, respectively (see refs 8, 16, 29, 59 for discussion of advantages and limitations of the PIP_25_ approach). Most recently, Smik *et al*.^62^ introduced a HBI–III alkene as phytoplankton biomarker replacing the sterols in the PIP_25_ calculation. This modified PIP_25_ approach is far less dependent on the balance factor *c* and based on biomarkers from the same group of compounds (that is, HBIs) with more similar diagenetic sensitivity, certainly an important improvement for paleo-sea-ice reconstructions and comparison of records from different Arctic areas.

Our reconstruction of SST is based on long-chain C_37_ alkenones synthesized by haptophyte algae^63^. The C_37:3_- and C_37:2_-alkenones were present in all samples, whereas the C_37:4_-alkenone was not found. For alkenone (C_37:2_ and C_37:3_) analysis, extraction of additional 6 g of freeze-dried sediment was carried out using the ASE method under same conditions as decribed above but with dichloromethane as the solvent. The separation of compounds was carried out by open column chromatography using 5 ml *n*-hexane, followed by 5 ml *n*-hexane:dichloromethane (1:1, v/v) and 5 ml dichloromethane for eluation of the alkenones. As internal standard *n*-C_36:0_ (10 μg per sample) was added before any analytical treatment. The alkenones were analysed by GC. Individual alkenone (C_37:3_ and C_37:2_) identification is based on retention time and the comparison with an external standard (Supplementary Fig. 7). To exclude a possible coeluation of the alkenones with other compounds, the extracts were measured first as total extract, second after additional column cleaning with dichloromethane and third after saponification^64^. The instrument stability has been continuously controlled by re-runs of an external alkenone standard (extracted from cultures of *Emiliania huxleyi* with known growth temperature) during the analytical sequences. The range of the total analytical error calculated by replicate analyses is <0.4 °C.

**Figure 4:**
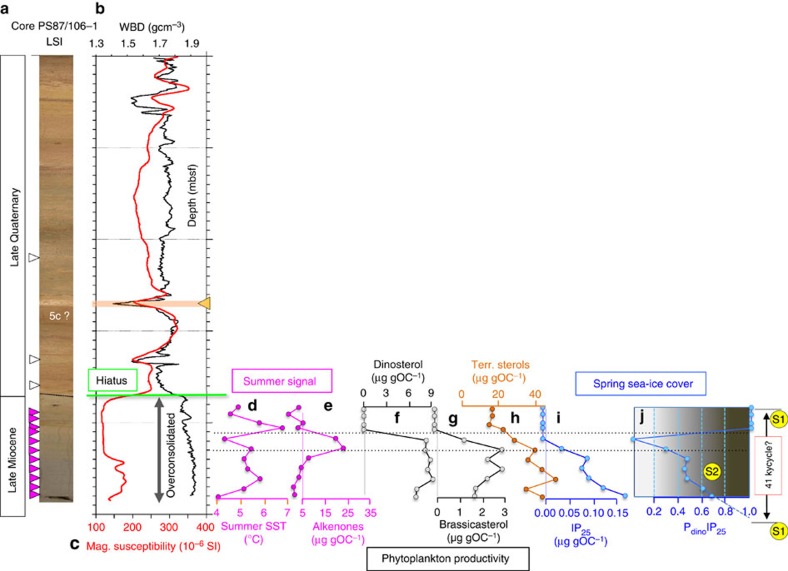
Proxy evidence from Core PS87/106 for late Miocene Arctic Ocean climate conditions. (**a**) Line-scan image (LSI), (**b**) WBD, (**c**) MS, (**d**) alkenone-based SST, (**e**) concentrations of alkenones, (**f**) dinosterol and (**g**) brassicasterol as proxy for primary productivity, (**h**) concentrations of terrigenous sterols (sum of campesterol and β-sitosterol), (**i**) concentrations of sea-ice proxy IP_25_ and (**j**) sea-ice index P_dino_IP_25_ (for data see Supplementary Table 2). Purple and open triangles indicate the presence or absence of alkenones, respectively. Green line marks depth of the hiatus. Below the hiatus, sediments are overconsolidated. Zero or near-zero concentrations of IP_25_ and phytoplankton biomarkers are indicative for a closed (spring) sea-ice cover (P_dino_IP_25_ is indetermable and set to ‘1'; see Fig. 1 and Methods for further details). PIP_25_ values are used to identify low (0–20%), common (20–50%), ice-edge (50–70%) and extended (>70%) sea-ice concentrations (see Fig. 1). The section below the hiatus represents about one cold (‘glacial')–warm (‘interglacial') cycle. S1 and S2 indicate two scenarios of different sea-ice concentrations (that is, maximum and transitional/stable ice edge, respectively) within this climate cycle. The interval between the two dashed horizontal lines marks period with minimum sea-ice cover. Using mean sedimentation rates of ∼3.2 cm ky^−1^ (as calculated independently from close-by gravity cores^50^ and seismic data^51^), the duration of the climate cycle is ∼40 ky.

**Figure 5:**
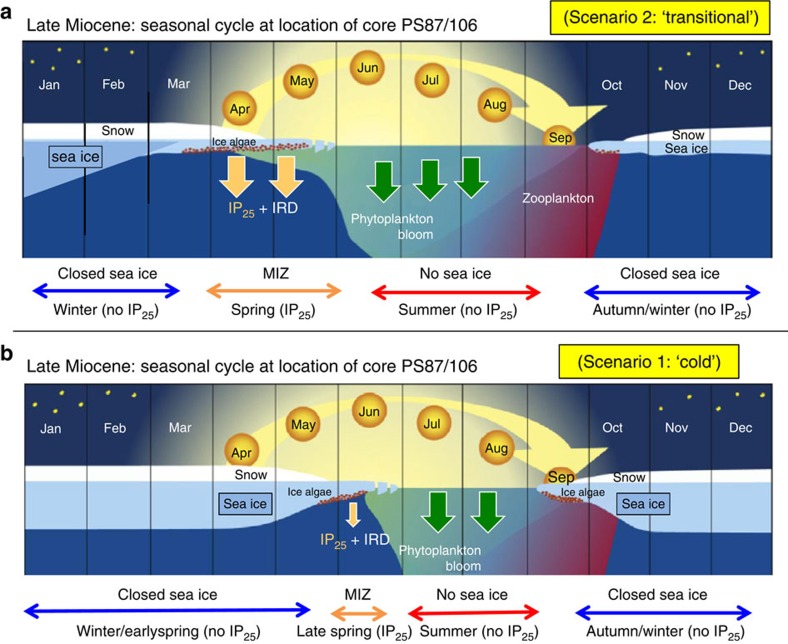
Schematic illustrations of the seasonal sea-ice cycle during the late Miocene. The seasonal sea-ice cycle and related principal processes controlling productivity and carbon flux at location of central Arctic Ocean Core PS87/106 during the late Miocene are shown for two different scenarios. (**a**) Scenario 2 (‘warmer/transitional situation')=extended period of spring sea-ice algae productivity and increased IP_25_ and phytoplankton biomarker fluxes. (**b**) Scenario 1 (‘cold situation')=restricted period of late spring sea-ice algae productivity and very reduced fluxes (almost to zero) of IP_25_ and spring phytoplankton biomarkers; *cf*., Fig. 1). MIZ, Marginal Ice Zone, that is, ice-edge situation. The dark period, height of the sun and changing thickness of snow and ice over the year, as well as phytoplankton, zooplankton and sea-ice productivity are shown. IP_25_ values for the different seasons are indicated (after ref. 72, supplemented).

**Figure 6:**
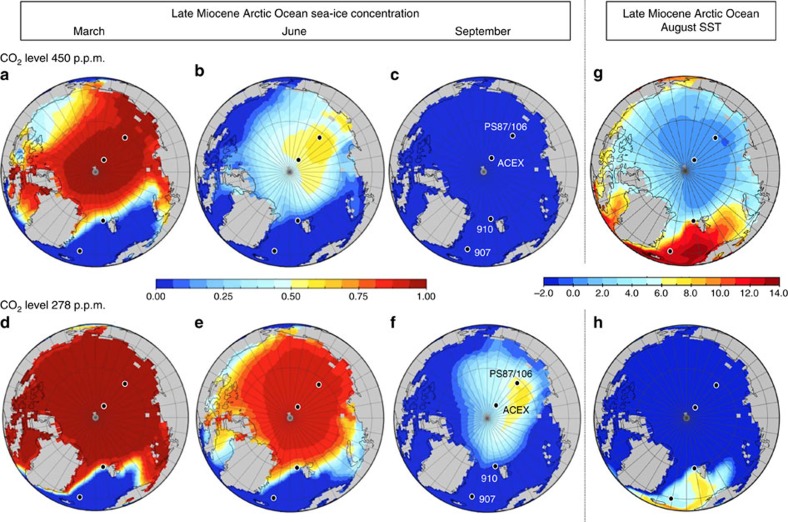
Late Miocene Arctic Ocean climate simulations for high and low pCO_2_ levels using a coupled atmosphere–ocean general circulation model. (**a**–**c**,**d**–**f**) Sea-ice concentrations for March, June and September at 450 and 278 p.p.m. CO_2_ levels, respectively^32^. (**g**,**h**) Late Miocene August SST at 450 and 278 p.p.m. CO_2_ levels, respectively.

**Figure 7:**
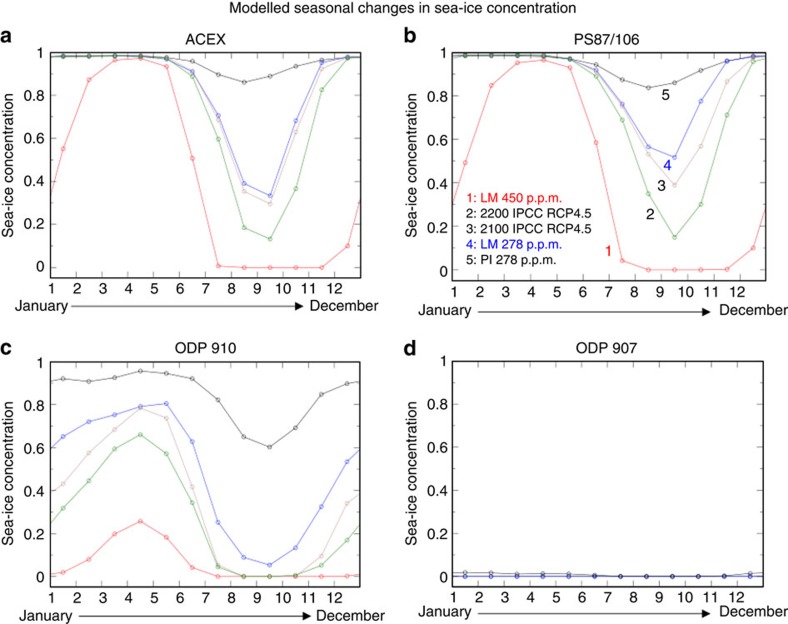
Modelled monthly sea-ice concentration at the four different core locations. (**a**) ACEX Site, (**b**) Core PS87/106, (**c**) ODP Site 910 and (**d**) ODP Site 907. Climatological monthly mean sea-ice concentrations for late Miocene conditions with atmospheric CO_2_ concentrations of 450 p.p.m. (red curve) and 278 p.p.m. (blue curve), modelled sea-ice concentration for preindustrial conditions (black curve) with an atmospheric CO_2_ concentration of 278 p.p.m.^32^, as well as future conditions (monthly mean 2,085–2,115, brown curve; monthly mean 2,185–2,215, green curve) following the Intergovernmental Panel on Climate Change (IPCC) medium stabilization scenario RCP4.5 (Gierz *et al*.^40^) are shown.

**Figure 8:**
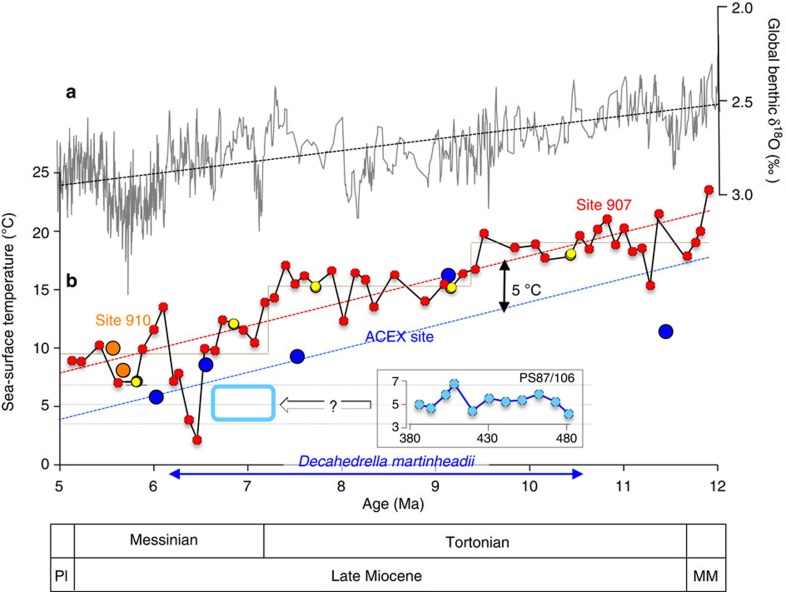
Global climate (benthic δ^18^O) stack and (sub-) Arctic SST records for the Late Miocene time interval. (**a**) Global benthic δ^18^O stack from ref. 43. (**b**) Late Miocene (Messinian/Tortonian) alkenone-based *U*^*k*'^_37_-SST of Site 907 (yellow circles)^27^ and this study (red circles; Supplementary Table 5), TEX_86_-SST values of Site 910 (orange circles)^12^ and alkenone-based *U*^*k*'^_37_-SST of the ACEX Site (blue circles; this study/Supplementary Table 2). The ages of the five ACEX samples are based on ^10^Be stratigraphy^73^. General cooling trends in the 907 SST record (stippled red line), the ACEX SST record (stippled blue line, assuming a parallel SST trend to Site 907 with an offset of about 5 °C) and the benthic δ^18^O record (stippled black line) are indicated. The alkenone-based *U*^*k*'^_37_-SST of Core PS87/106 are plotted versus depth (centimetres below seafloor) (*cf*., Supplementary Table 2). The stratigraphic range of the acritarch *D. martinheadii* in the sub-Arctic/Arctic realm is indicated^24^,^25^. As the central Arctic Ocean ACEX and the PS87/106 SST values should be similar, the PS87/106 record probably repesents a time span within the upper Tortonian to lower Messinian (light blue box). Pl, Pliocene; MM, Middle Miocene.

For calculation of SST, we used the simplified 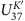 Index^63^:


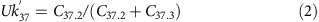



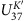
 was converted to SST according to the World Ocean core top versus annual temperature calibration 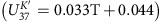 (ref. 64), the calibration most often used in the literature. Resulting SSTs vary between 4.2 and 6.7 °C (SST-1; Fig. 4 and Supplementary Table 2). For the central Arctic Ocean, these SSTs certainly have to be interpreted as summer SSTs (instead of annual mean) due to the darkness during late autumn to winter (*cf*., Fig. 5). In addition, we also have used the Müller *et al*.^64^ calibration versus summer SST (SST-2), the Sikes *et al*.^65^ calibration versus summer SST obtained from the polar Southern Ocean (SST-3) and the Prahl and Wakeham^63^ calibration obtained from cultural experiments (SST-4). Whereas the SST-4 values are more or less the same as the SST-1 values, the SST-2 and SST-3 values (calculated as ‘summer SST') are higher and vary between 6 and 9 °C (Supplementary Table 2). Based on these results, we interpret our late Miocene summer SSTs of ∼4–7 °C (mean of 5.3 °C) more as minimum values. For the Müller *et al*.^64^ calibration, the standard error is reported as ±0.050 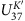
units or ±1.5 °C for the entire temperature range from 0 to 27 °C. In the lower temperature range <10 °C, however, the scatter of the 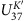 values is significantly higher than the mean. Thus, one should not overinterpret the SST variability between 4 and 7 °C. In any case and independently of the calibration approach, summer SSTs were significantly higher than zero, preventing sea-ice formation during summer.

The results of the biomarker analyses (that is, alkenones, selected sterols, SSTs, IP_25_ and PIP_25_) carried out on samples from various PS87 sediment cores, are listed in Supplementary Table 2. All biomarker data (expressed in μg gOC^−1^ and μg gSediment^−1^) are available online at http://dx.doi.org/10.1594/PANGAEA.855509.

### Model simulation

For the investigation of the late Miocene Arctic climatic conditions (that is, climatological sea-ice cover and SST) we have re-analysed Miocene climate simulations^32^,^39^. The simulations have been performed with a coupled AOGCM. The atmosphere model component ECHAM5 (ref. 66) was used at T31 resolution (∼3.75°) with 19 vertical levels. The ocean component MPI-OM^67^, including the dynamics of sea ice formulated using viscous-plastic rheology^68^, has an average horizontal resolution of 3° × 1.8° with 40 uneven vertical layers. This modelling approach has been used and evaluated for investigations of the Miocene climate^32^,^39^. For the re-analyses, we have used data from two model runs with the same late Miocene set-up^32^, except different atmospheric CO_2_ concentrations. One simulation is based on a CO_2_ concentration of 278 p.p.m.^32^ and one uses a CO_2_ concentration of 450 p.p.m.^39^. For further details of the AOGCM model configuration and the boundary conditions, we refer the reader to refs 32, 39.

### Sediment load and compaction experiments

The preconsolidation stress of a geological sample experienced in the past can be assessed by incrementally loading the specimen in a uniaxial deformation apparatus (so-called ‘oedometer'), where deviations in the settling behaviour can be converted to the thickness of the missing overburden. We used this approach to estimate the thickness of sediment removed at prominent unconformities observed in some of our studied sediment cores (Fig. 4 and Supplementary Figs 2 and 8).

We conducted our experiments using a combined GIESA oedometer—direct shear apparatus in which both uniaxial compression tests and shear tests can be conducted in consecutive steps^69^ (Supplementary Fig. 8b). The sample cell is a cylindrical volume within a stack of two steel plates. If desired, relative displacement of the plates enforces simple shear deformation in the sample, to measure undrained shear strength. Porous metal frits allow fluid communication with an open pore fluid reservoir (containing distilled water) and dissipation of excess pore pressure. Normal load is applied to the sample with a vertical ram and shear is induced by holding the upper plate fixed, while the lower plate is driven horizontally.

For the experiments in this study, we only used the consolidation function of the system. As is done routinely in such tests^70^, we loaded each sample incrementally by starting with applied normal stresses of 10 kPa. This value was assumed to be below the *in situ* stress the samples had experienced before. After 24 h, the normal load is doubled. Tests were run over many days until the desired maximum normal load was reached (10,240 kPa for Core PS87/096 and 5,120 kPa for Core PS87/106). The initial water content of each sample was taken from an aliquot before the specimen was mounted into the GIESA oedometer. We measured the wet weight of the aliquot, subjected it to 48 h of gentle drying in an oven (60 °C) and then measured the dry weight, to calculate the void ratio *e* (where *e*=volume of voids/volume of solids).

Oedometer results are plotted as effective normal stress versus void ratio, the latter of which is derived from the settling (that is, change in sample thickness as determined using a vertical displacement transducer) after each loading increment. The graph of each experiment show a smooth function when *e* is plotted against normal stress, the latter on a logarithmic axis (Supplementary Fig. 8c). In an undeformed sample, the regular loading results in the so-called ‘virgin consolidation curve', whereas samples that were previously subject to loading usually deviate from the smooth curvature and show a distinct change in gradient of the graph. The high-stress end of the graph is generally linear and serves to assess the maximum preconsilidation stress the sample has experienced following the procedure first established by A. Casagrande^69^.

The stress measurements are then used to the thickness (*h*) of the overburden based on the effective load (*h*=preconsolidation stress/(bulk density of the sample−density of seawater) × *g*). The degree of overconsolidation of a given sample is then calculated as the ratio between preconsolidation stress and normal stress at that depth below the seafloor.

We have tested one pair of samples in cores PS87/096 and PS87/106, respectively, and loaded these samples to at least 5,120 kPa effective stress. Our results attest that the samples taken above the hiatuses are normally consolidated, while those underneath the discontinuity are overconsolidated. In Core PS87/096, the estimated preconsolidation stress below the hiatus is 590 kPa, which amounts to the removal of a 80-m-thick sediment package (Supplementary Fig. 8c). In Core PS87/106, the preconsolidation stress is between 320 and 480 kPa, which corresponds to 48 and 65 m of overburden, respectively (*cf*., Fig. 4).

For Core PS87/096, the calculated thickness of removed sediments are more or less identical to those estimated from the ages of the sediments above and below the hiatus (that is, ∼100 ka and <2.5 Ma, respectively) and mean sedimentation rates of 3.2 cm ky^−1^ (refs 50, 71), resulting in a maximum sediment removal of 80 m (Supplementary Fig. 8a).

## Additional information

**How to cite this article:** Stein, R. *et al*. Evidence for ice-free summers in the late Miocene central Arctic Ocean. *Nat. Commun.* 7:11148 doi: 10.1038/ncomms11148 (2016).

## Supplementary Material

Supplementary Information Supplementary Figures 1-8, Supplementary Tables 1-5, Supplementary Note 1 and Supplementary References.
