# Supplementary material for: Evidence for ice-free summers in the late Miocene central Arctic Ocean
Source: Nat Commun. 2016 Apr 4;7:11148. doi: 10.1038/ncomms11148 (PMC4822014; doi:10.1038/ncomms11148)
Supplement: Supplementary Information — Supplementary Figures 1-8, Supplementary Tables 1-5, Supplementary Note 1 and Supplementary References. [file ncomms11148-s1.pdf]

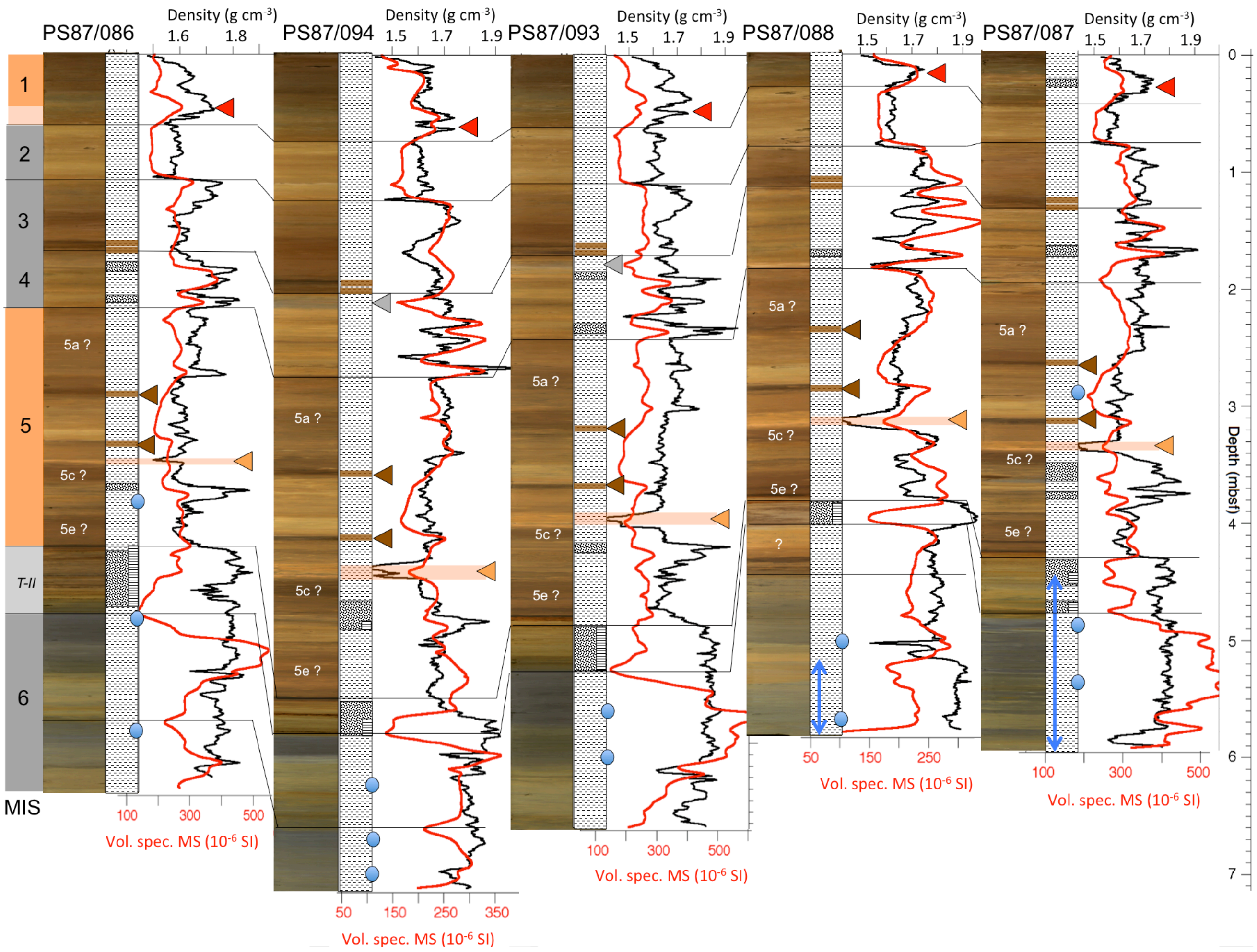

Supplementary Figure 1. Lithostratigraphy and age model of selected PS87 sediment cores from Lomonosov Ridge (Part 1: continuous sedimentary records, no hiatus). Lithostratigraphy and age model are based on main lithologies, wet-bulk density, magnetic susceptibility and color images. Prominent features identified and used for correlation are marked by triangles. MIS 6 to 1 are identified by correlation with dated sediment cores from adjacent areas[1]. Blue circles indicate depth location of samples used for biomarker studies (cf., Supplementary Table 2). Blue arrows at cores PS87/087 and PS87/088 indicate intervals with more consolidated/overconsolidated sediments. Locations of cores are listed in Supplementary Table 1 and shown in Figure 3.

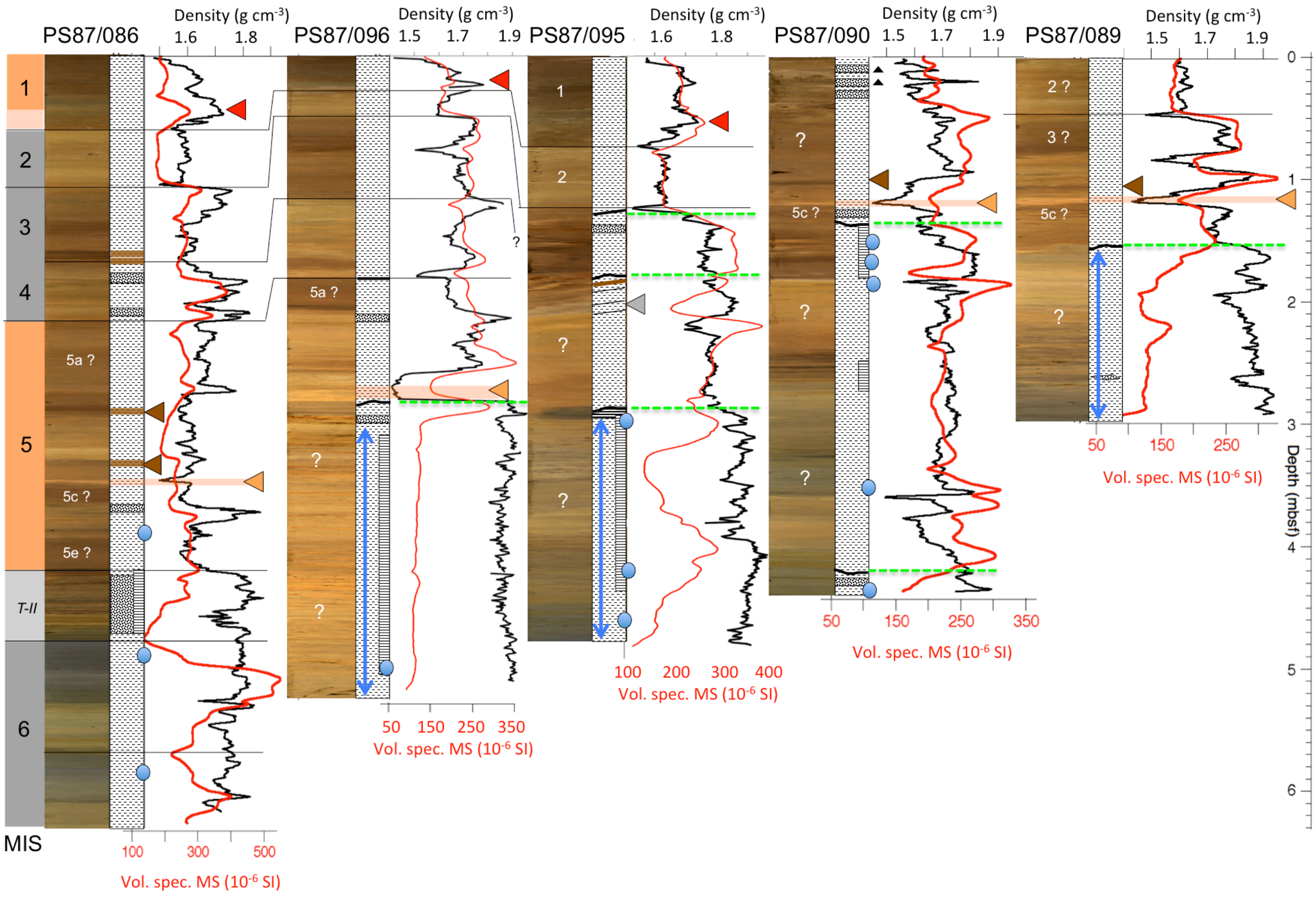

Supplementary Figure 2. Lithostratigraphy and age model of selected PS87 sediment cores from Lomonosov Ridge (Part 2: discontinuous sedimentary records with unconformities/hiatuses). Lithostratigraphy and age model are based on main lithologies, wet-bulk density, magnetic susceptibility and color images. Prominent features identified and used for correlation are marked by triangles. Stippled green lines indicate unconformities/hiatuses[1]. Blue circles indicate depth location of samples used for biomarker studies (cf., Supplementary Table 2). Blue arrows at cores PS87/096, PS87/095 and PS87/089 indicate intervals with more consolidated/overconsolidated sediments. Locations of cores are listed in Supplementary Table 1 and shown in Figure 3.

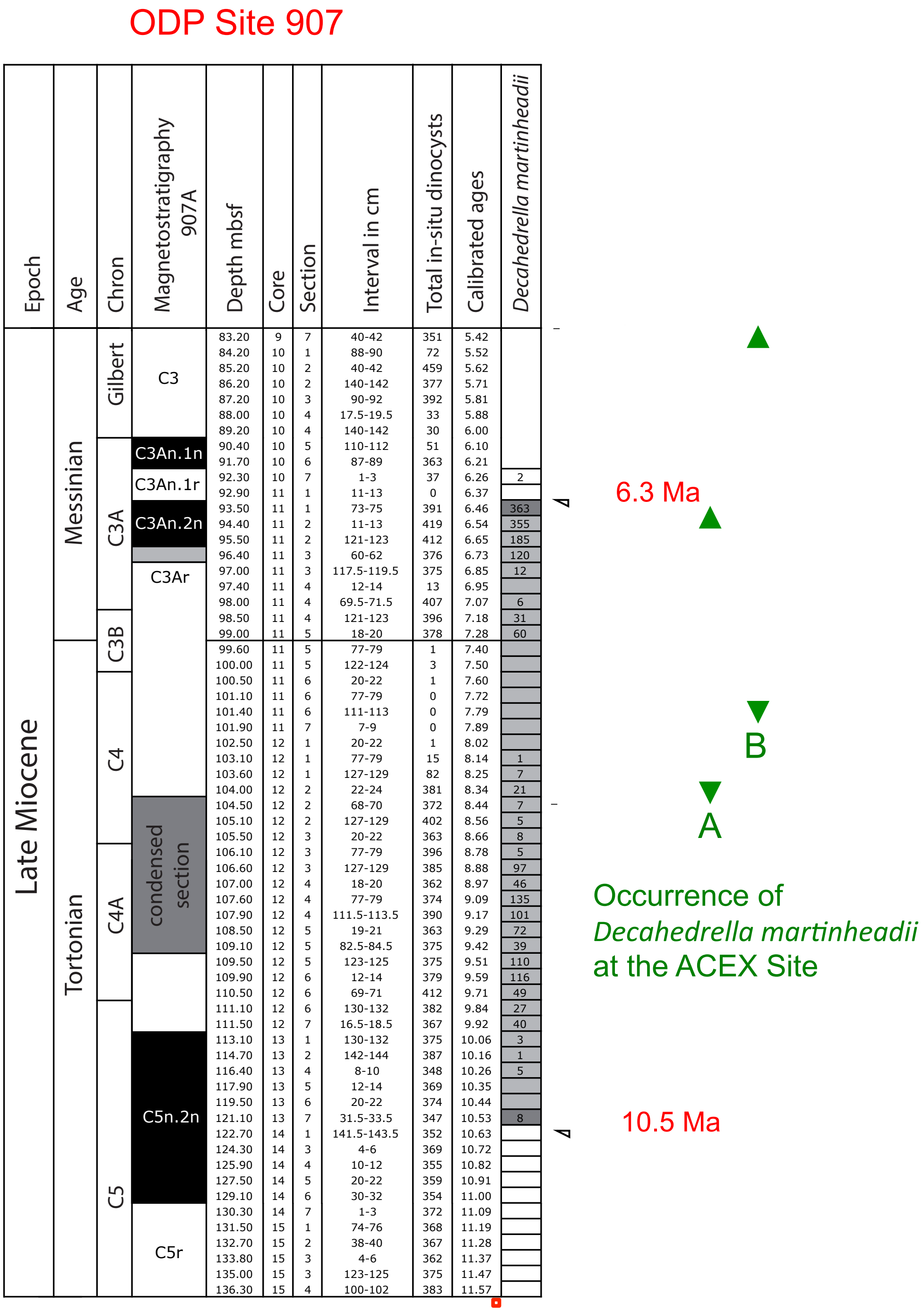

Depth mbsf	Core	Section	Interval in cm	Total in-situ dinocysts	Calibrated ages	*Decahedrella martinheadii*
83.20	9	7	40-42	351	5.42	
84.20	10	1	88-90	72	5.52	
85.20	10	2	40-42	459	5.62	
86.20	10	2	140-142	377	5.71	
87.20	10	3	90-92	392	5.81	
88.00	10	4	17.5-19.5	33	5.88	
89.20	10	4	140-142	30	6.00	
90.40	10	5	110-112	51	6.10	
91.70	10	6	87-89	363	6.21	
92.30	10	7	1-3	37	6.26	2
92.90	11	1	11-13	0	6.37	
93.50	11	1	73-75	391	6.46	363
94.40	11	2	11-13	419	6.54	355
95.50	11	2	121-123	412	6.65	185
96.40	11	3	60-62	376	6.73	120
97.00	11	3	117.5-119.5	375	6.85	12
97.40	11	4	12-14	13	6.95	
98.00	11	4	69.5-71.5	407	7.07	6
98.50	11	4	121-123	396	7.18	31
99.00	11	5	18-20	378	7.28	60
99.60	11	5	77-79	1	7.40	
100.00	11	5	122-124	3	7.50	
100.50	11	6	20-22	1	7.60	
101.10	11	6	77-79	0	7.72	
101.40	11	6	111-113	0	7.79	
101.90	11	7	7-9	0	7.89	
102.50	12	1	20-22	1	8.02	
103.10	12	1	77-79	15	8.14	1
103.60	12	1	127-129	82	8.25	7
104.00	12	2	22-24	381	8.34	21
104.50	12	2	68-70	372	8.44	7
105.10	12	2	127-129	402	8.56	5
105.50	12	3	20-22	363	8.66	8
106.10	12	3	77-79	396	8.78	5
106.60	12	3	127-129	385	8.88	97
107.00	12	4	18-20	362	8.97	46
107.60	12	4	77-79	374	9.09	135
107.90	12	4	111.5-113.5	390	9.17	101
108.50	12	5	19-21	363	9.29	72
109.10	12	5	82.5-84.5	375	9.42	39
109.50	12	5	123-125	375	9.51	110
109.90	12	6	12-14	379	9.59	116
110.50	12	6	69-71	412	9.71	49
111.10	12	6	130-132	382	9.84	27
111.50	12	7	16.5-18.5	367	9.92	40
113.10	13	1	130-132	375	10.06	3
114.70	13	2	142-144	387	10.16	1
116.40	13	4	8-10	348	10.26	5
117.90	13	5	12-14	369	10.35	
119.50	13	6	20-22	374	10.44	
121.10	13	7	31.5-33.5	347	10.53	8
122.70	14	1	141.5-143.5	352	10.63	
124.30	14	3	4-6	369	10.72	
125.90	14	4	10-12	355	10.82	
127.50	14	5	20-22	359	10.91	
129.10	14	6	30-32	354	11.00	
130.30	14	7	1-3	372	11.09	
131.50	15	1	74-76	368	11.19	
132.70	15	2	38-40	367	11.28	
133.80	15	3	4-6	362	11.37	
135.00	15	3	123-125	375	11.47	
136.30	15	4	100-102	383	11.57	

Supplementary Figure 3. Stratigraphy of ODP Hole 907A. Raw counts and stratigraphic ranges of the acritarch Decahedrella martinheadii in ODP Hole 907A[6, 7]. Light shading indicates total stratigraphic range, and dark shading indicates the LO and HO respectively. Also shown is the magnetic polarity stratigraphy of Hole 907A[14]. Total in-situ counts refers to the total in-situ dinocysts plus the acritarch *D. martinheadii* (after Schreck et al. [6]). Green arrows indicate the stratigraphic range of *D. martiheadii* in IODP/ACEX Hole M2A based on A) the age model of Backman et al. [15], and B) the age model of Frank et al. [16] (from Matthiessen et al. [5]).

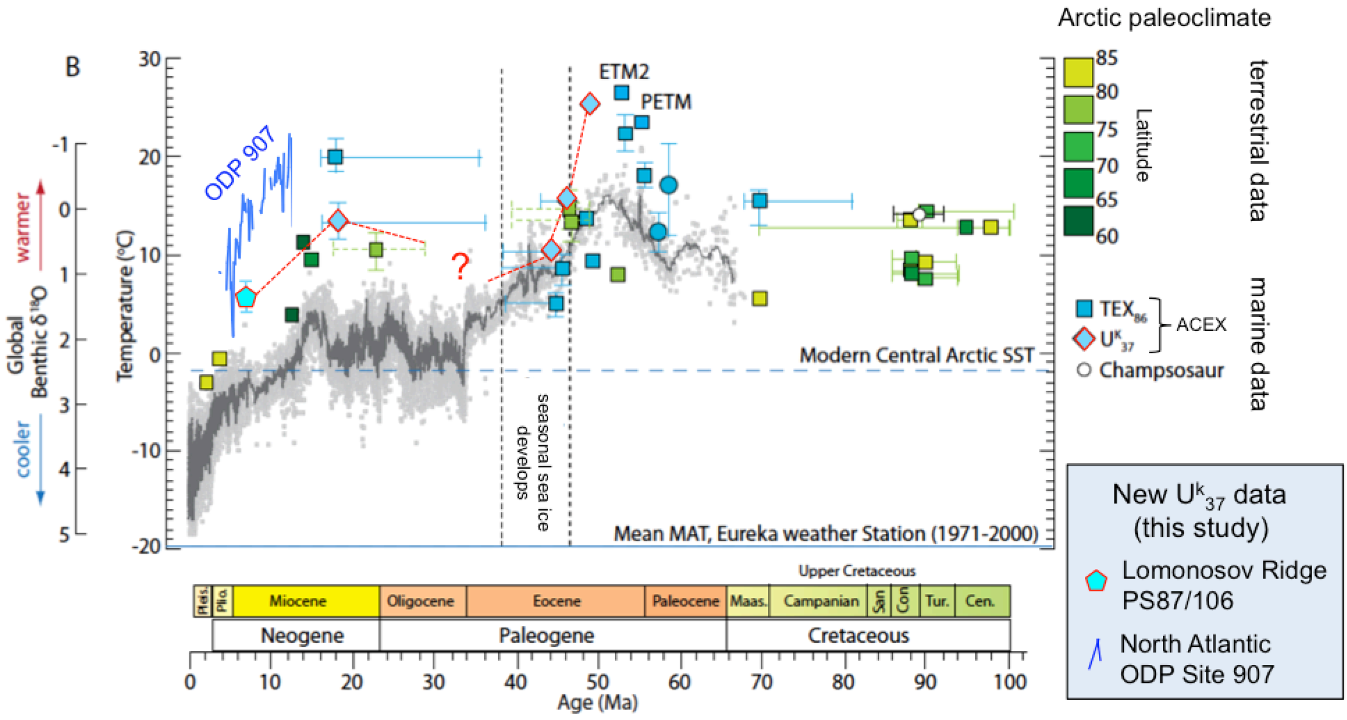

Supplementary Fig. 4. Compiled air and sea-surface temperature, based on data from terrestrial (green symbols) and marine (blue symbols) records and overlain on the global benthic $\delta^{18}O$ stack[17] (Figure from ref. 18, supplemented). The terrestrial data are color coded according paleolatitude (references of data sources are listed in ref. 18. TEX_{86}-based SST data (data source: refs. 19-21) and U^{k}_{37}-based SST data (refs. 22, 23) from the ACEX site are marked as blue squares and blue rhombs, respectively. In addition, the new U^{k}_{37}-based SST data of Core PS87/106 and ODP Site 907 are shown (for data see Supplementary Tables 2 and 5). Stippled red line connect the U^{k}_{37}-based SST data points from Core PS87/106 and the ACEX site; red question mark highlights data gap between about 44 and 18 Ma. Further details and references ref. 18.

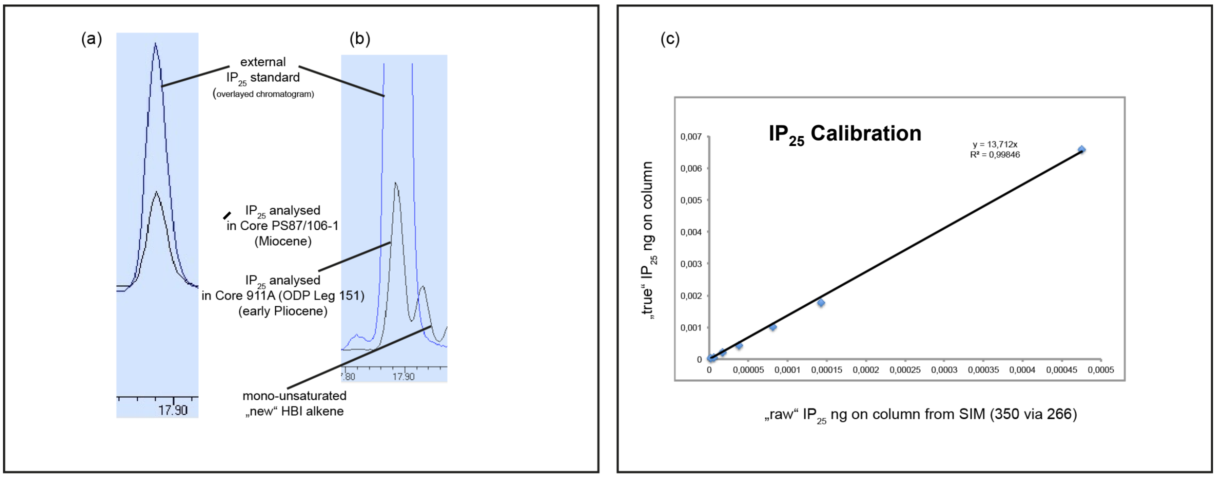

Supplementary Figure 5. Identification and quantification of IP_{25} (a) Selected-ion monitoring chromatogram (SIM mode) of ion m/z 350 of the extrernal IP_{25} standard and IP_{25} analysed from Core PS87/106-1 (overlayed chromatograms); (b) Selected-ion monitoring chromatogram of ion m/z 350 of the external IP_{25} standard (blue curve) and IP_{25} and the mono-unsaturated HBI alkene (according to ref. 24) from ODP Hole 911A (early Pliocene; cf. ref. 25 and own unpubl. data) and (c) Calibration curve for quantification of IP_{25} („raw“: ration of 350/266 without concideration of the different responses of the ions; „true“: quantification under consideration of the different responses of the ions).

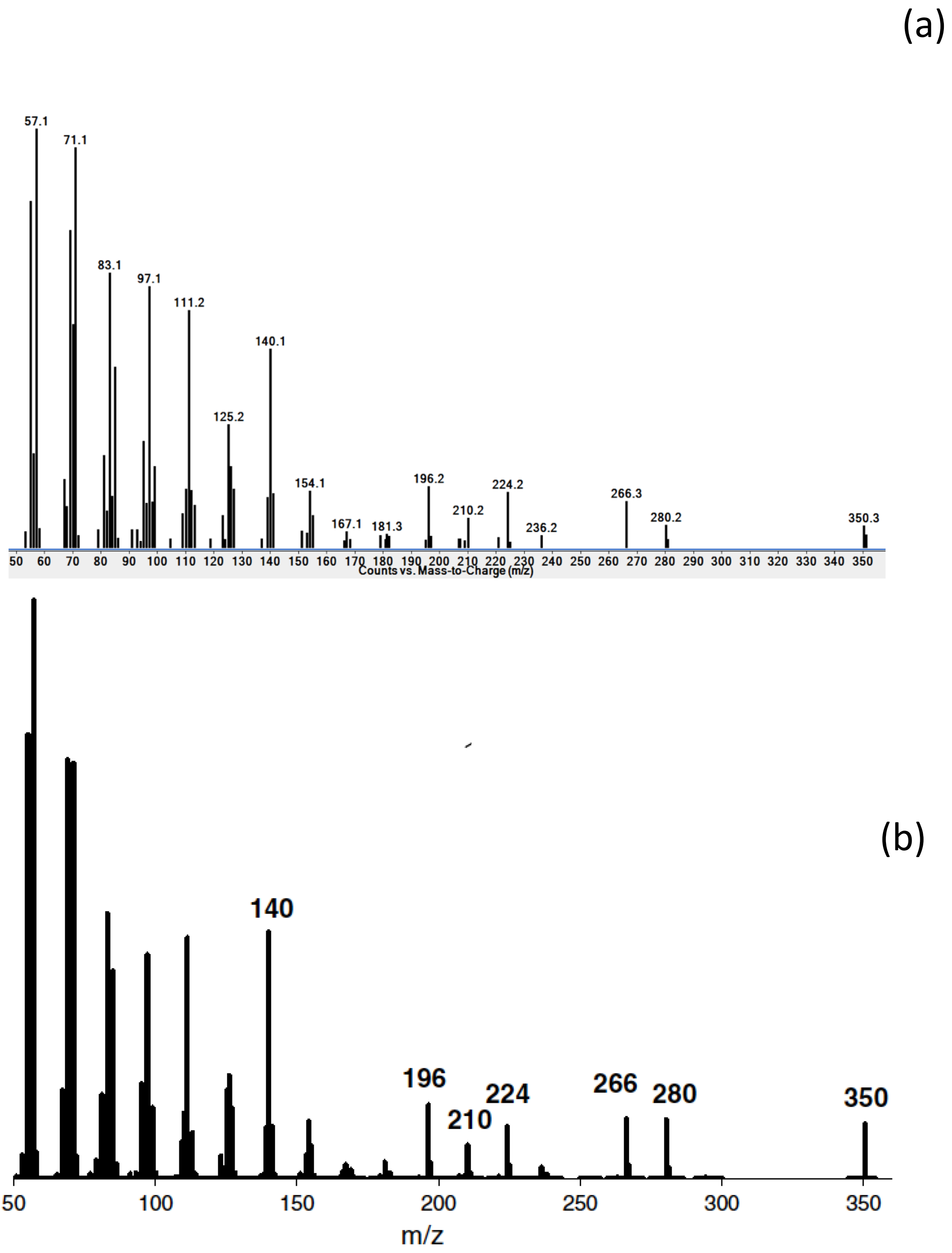

Supplementary Figure 6. (a) Mass spectrum of IP_{25} from PS87/106-1 478 cm (this study) and (b) mass spectrum of IP_{25} published by Belt et al.[26].

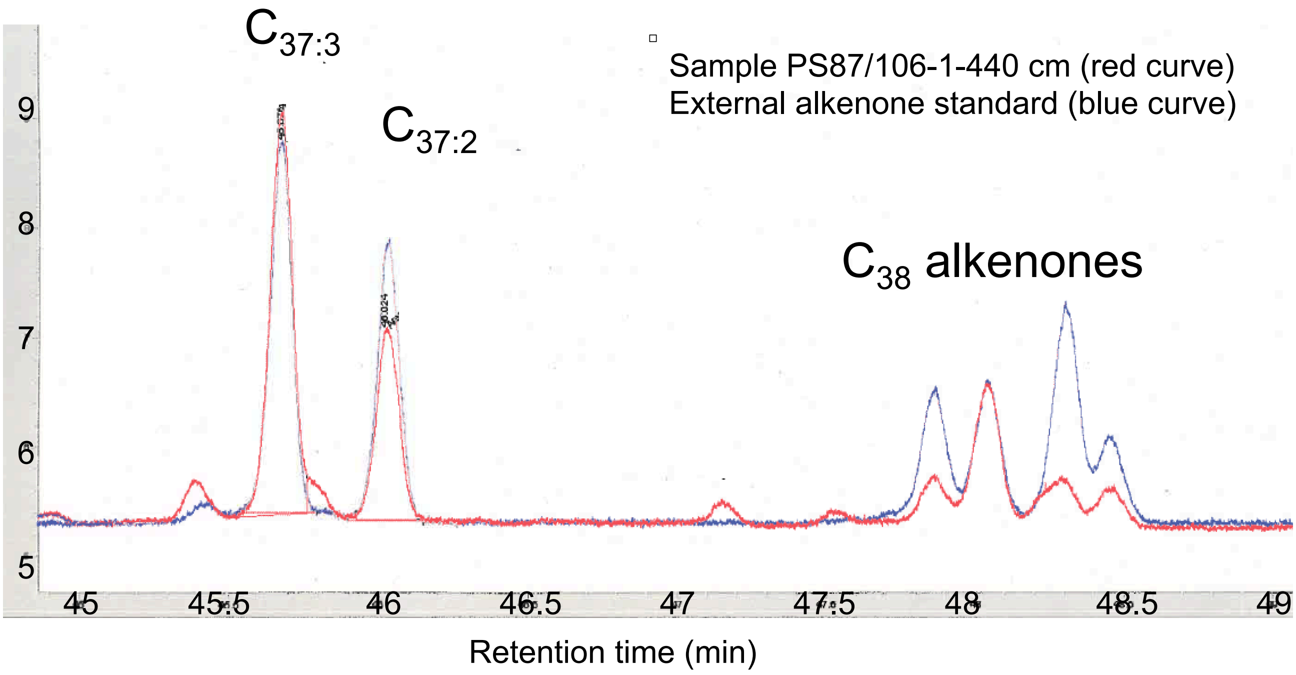

Supplementary Figure 7. GC chromatograms of alkenone standard (from culture experiments, blue curve) and sample PS87/106-1-440 cm (red line) of this study.

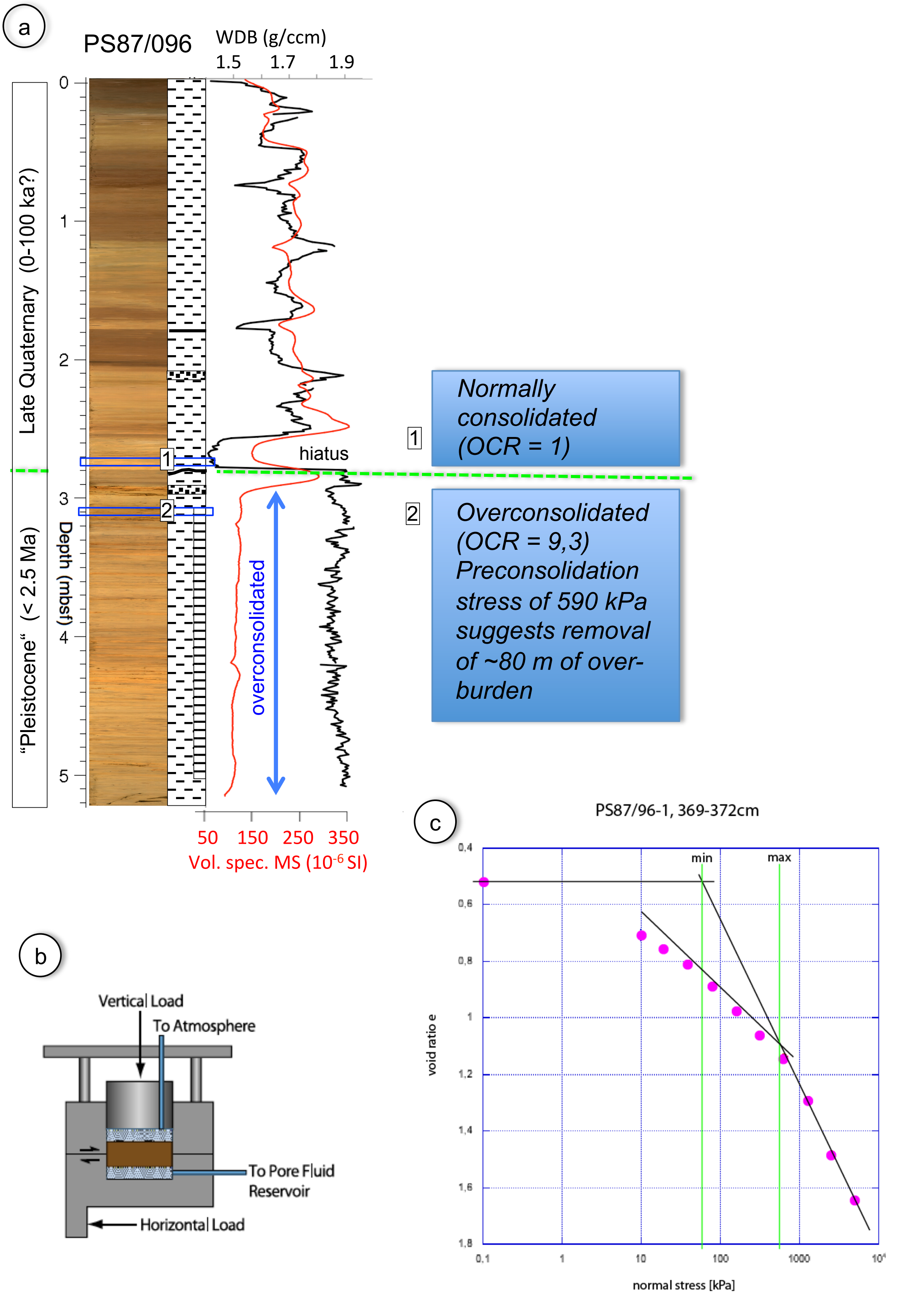

Supplementary Figure 8. Results of sediment load and compaction experiments. **a.** Main lithologies, wet-bulk density, magnetic susceptibility and color images of Core PS87/096 and depth of major hiatus (stippled green line). **b.** Schematic illustration of the oedometer used in our compaction experiment[27]. **c.** Oedometer results plotted as effective normal stress vs. void ratio; for further explanation see Methods.

Supplementary Table 1. Chronology of PS87 sediment cores based on microfossil assemblages in core catcher (CC) samples. a. Core location (latitude and longitude), water depth (WD), core catcher (CC) depth in cm below sea floor (cmsf) (ref. 1) as well as age estimate based on agglutinated benthic foraminifers (ABF) (correlation with biostratigraphic records from the North Atlantic and Arctic Ocean[2,3]), are listed (for details see Chapter 10.8 in ref. 1). **b.** Occurrence (counts) of the acritarch *Decahedrella martinheadii*, age marker for the late Miocene, 10.5-6.3 Ma (ref. 4-7), in samples from Core PS87/106; sample depth in cm below sea floor (cmsf).

a) Core catcher samples

Core	Lat	Long	WD(m)	CC (cmbsf)	ABF (CC)
**Transect 1**					
PS87/086-3	81°13.04'N	141°23.02'E	902	629	Pleistocene
PS87/096-1	81°12.73'N	141°20.03'E	1071	523	Pleistocene
PS87/095-1	81°12.64'N	141°19.22'E	1123	478	barren
PS87/094-1	81°12.57'N	141°18.48'E	1166	712	Late Miocene?
PS87/093-1	81°12.48'N	141°17.62'E	1214	673	Late Miocene?
PS87/090-1	81°12.37'N	141°16.89'E	1316	439	barren
PS87/089-1	81°12.35'N	141°16.49'E	1355	294	barren
PS87/088-1	81°12.31'N	141°16.40'E	1379	586	Early Pleistocene
PS87/087-1	81°12.28'N	141°15.86'E	1416	594	barren
**Transect 2**					
PS87/102-1	81°12.86'N	141°11.37'E	1334	678	barren
PS87/103-1	81°12.84'N	141°11.14'E	1367	353	Early Pleistocene
PS87/104-1	81°12.80'N	141°10.85'E	1414	317	barren
PS87/108-1	81°12.79'N	141°10.69'E	1439	129	Early Pleistocene
PS87/105-1	81°12.78'N	141°10.64'E	1453	88	Early Pleistocene
PS87/106-1	81°12.76'N	141°10.47'E	1472	486	barren
PS87/107-1	81°12.75'N	141°10.25'E	1495	556	barren

b) Acritarch data from Core PS87/106-1

Core	Lat	Long	WD	cmbsf	*D. martinheadii*
PS87/106-1	81°12.76'N	141°10.47'E	1472	358	0
				388	0
				395	0
				404	0
				409	0
				420	13
				430	32
				440	51
				450	22
				460	5
				470	8
				477	11

Supplementary Table 2. Total organic carbon (TOC) and biomarker (alkenones, sterols, and IP_{25}) data from selected PS87 sediment cores and the ACEX Site, and intepretation of the biomarker data in terms of sea-ice coverage for spring, summer, and winter. PIP_{25} values were calculated using IP_{25} and brassicasterol and dinosteol concentrations ($P_{bras}IP_{25}$ and $P_{dino}IP_{25}$, respectively). Alkenone-based sea-surface temperatures (SSTs) determined in the Miocene sediments of Core PS87/106 and the ACEX Site were calculated using different calibrations – SST-1 and SST-2 (ref. 8), SST-3 (ref. 9), and SST-4 (ref. 10) (for further explanation see text of main manuscript).

Core	Core depth (mbsf)	TOC (%)	SST-1 (°C)	SST-2 (°C)	SST-3 (°C)	SST-4 (°C)	UK'37	Alkenones (µg/gTOC)	P(dino)IP25	P(brass)IP25	IP25 (µg/gTOC)	Brassicasterol (µg/gTOC)	Dinosterol (µg/gTOC)	Campesterol (µg/gTOC)	ß-sitosterol (µg/gTOC)	Sea ice Spring	Sea ice Summer	Sea ice Winter
PS87/086	3,86	0,11	-	-	-	-	-	0,000	-	-	0,000	0,000	0,000	0,900	7,787			
PS87/086	4,86	0,66	-	-	-	-	-	0,000	-	-	0,530	4,506	3,815	16,182	99,113			
PS87/086	5,81	0,37	-	-	-	-	-	0,000	-	-	0,000	1,276	3,199	4,576	25,771			
PS87/087	2,88	0,23	-	-	-	-	-	0,000	-	-	0,000	present	0,000	1,391	13,084			
PS87/087	4,88	0,47	-	-	-	-	-	tr	-	-	0,778	4,869	5,256	21,332	134,659			
PS87/087	5,36	0,22	-	-	-	-	-	0,000	-	-	0,000	present	0,000	1,233	67,707			
PS87/088	5,01	0,29	-	-	-	-	-	tr	-	-	0,000	present	3,136	2,419	21,098			
PS87/088	5,71	0,22	-	-	-	-	-	0,000	-	-	0,000	present	present	0,997	9,322			
PS87/090	1,40	0,10	-	-	-	-	-	tr	-	-	0,000	0,000	0,000	1,193	8,221			
PS87/090	1,69	0,10	-	-	-	-	-	0,000	-	-	0,000	0,000	0,000	0,000	9,122			
PS87/090	1,81	0,13	-	-	-	-	-	0,000	-	-	0,000	0,000	0,000	0,165	10,550			
PS87/090	3,50	0,27	-	-	-	-	-	0,000	-	-	0,000	present	1,698	1,696	12,835			
PS87/090	4,30	0,21	-	-	-	-	-	0,000	-	-	0,000	0,000	1,838	1,623	7,396			
PS87/093	5,60	0,57	-	-	-	-	-	0,000	-	-	0,540	3,930	5,955	21,784	138,758			
PS87/093	6,00	0,29	-	-	-	-	-	0,000	-	-	0,000	present	2,087	3,522	16,536			
PS87/094	6,25	0,20	-	-	-	-	-	0,000	-	-	0,000	0,000	3,592	6,282	37,886			
PS87/094	6,70	0,34	-	-	-	-	-	0,000	-	-	0,079	2,192	4,692	5,680	36,850			
PS87/094	7,00	0,31	-	-	-	-	-	0,000	-	-	0,000	1,275	1,994	2,766	11,675			
PS87/095	2,95	0,11	-	-	-	-	-	0,000	-	-	0,000	0,000	0,000	0,000	2,958			
PS87/095	4,20	0,19	-	-	-	-	-	0,000	-	-	0,000	0,000	0,000	0,000	5,490			
PS87/095	4,60	0,34	-	-	-	-	-	0,000	-	-	0,000	present	2,762	2,273	18,017			
PS87/096	5,05	0,19	-	-	-	-	-	0,000	-	-	0,000	0,000	0,000	0,000	0,447			
PS87/106	2,40	0,20	-	-	-	-	-	0,000	-	-	0,000	present	present	0,268	9,250			
PS87/106	3,22	0,10	-	-	-	-	-	0,000	-	-	0,000	0,000	0,000	present	9,293			
PS87/106	3,58	0,12	-	-	-	-	-	0,000	-	-	0,000	0,000	present	present	5,854			
PS87/106	3,88	0,18	5,01	6,19	7,67	5,01	0,209	11,464	"1"	"1"	0,000	present	present	1,227	12,768			
PS87/106	3,95	0,19	4,70	5,83	7,36	4,66	0,198	5,313	"1"	"1"	0,000	0,000	present	1,363	12,701			
PS87/106	4,04	0,21	5,84	7,02	8,38	5,81	0,237	13,976	"1"	"1"	0,000	present	present	1,395	11,266			
PS87/106	4,09	0,24	6,75	7,93	9,17	6,70	0,267	11,032	"1"	"1"	0,000	present	present	2,473	16,329			
PS87/106	4,20	0,34	4,47	5,65	7,20	4,48	0,191	33,372	0	0	0,000	1,205	7,661	3,009	20,733			
PS87/106	4,30	0,41	5,52	6,71	8,11	5,51	0,226	37,923	0,28	0,22	0,035	2,744	7,537	5,175	27,636			
PS87/106	4,40	0,41	5,24	6,42	7,87	5,23	0,217	17,312	0,46	0,46	0,083	2,162	8,162	4,398	25,311			
PS87/106	4,50	0,40	5,38	6,56	7,98	5,36	0,221	12,948	0,44	0,37	0,073	2,752	7,823	5,164	30,753			
PS87/106	4,60	0,38	5,87	7,06	8,42	5,85	0,238	11,513	0,46	0,47	0,086	2,148	8,479	6,517	35,428			
PS87/106	4,70	0,38	5,23	6,41	7,86	5,23	0,217	8,383	0,59	0,60	0,113	1,676	6,493	3,326	25,577			
PS87/106	4,78	0,40	4,22	5,40	6,98	4,24	0,183	9,006	0,67	0,68	0,154	1,632	6,397	4,690	31,404			
302-2A-20-1-68	86,88	nd	5,50	6,60	8,00	5,40	0,224	nd	nd	nd	nd	nd	nd	nd	nd	?		?
302-2A-21-x		nd	8,93	10,11	11,07	8,81	0,339	nd	nd	nd	nd	nd	nd	nd	nd	?		?
302-2A-24-3-38	109,58	nd	9,00	10,20	11,20	8,90	0,342	nd	nd	nd	nd	nd	nd	nd	nd	?		?
302-2A-29-3-68	131,88	nd	15,80	17,00	17,00	15,50	0,565	nd	nd	nd	nd	nd	nd	nd	nd	?		?
302-2A-32-1-38	140,08	nd	10,70	11,90	12,60	10,50	0,397	nd	nd	nd	nd	nd	nd	nd	nd	?		?
																	sea-ice concentrations	
																30-70%	Ice-free	>80%

Supplementary Table 3. Modern (measured and modeled PI-278 ppm CO_2) mean August sea-surface temperatures (SST) and late Miocene Arctic Ocean mean August SST (proxy vs. modeling results). Modern August SST data from World Ocean Atlas (Source: http://odv.awi.de/en/data/ocean/world_ocean_ atlas_2013/). PI = preindustrial. For the Site 910 location, the modeled PI-278 SST is quite low in comparison to the measured SST value at that location. This difference can be explained by the very strong SST gradient close to an ice edge location and/or colder PI climate in comparison to today. Late Miocene (Messinian / Tortonian) alkenone-based $U^{k'}_{37}$-SST of Site 907 (ref. 7 and this study), and TEX_{86}-SST of Site 910 (ref. 11), proxy data from sites PS87/106 and ACEX as well as the modeling results for 450 and 278 ppm CO_2 scenarios from this study (for further explanation see text; for proxy data see Figs. 4 and 8).

Site	Latitude	Longitude	Modern	Modern	Late Miocene	Late Miocene	Late Miocene
			Measurement	*Model-PI 278*	*Proxy data*	*Model result-450*	*Model result-278*
Site 907	69°15'N	012°42'W	5 °C	7.5°C	9 °C / 18 °C	12.6 °C	8.7 °C
Site 910	80°16'N	006°35'E	2 °C	-1.9°C	9 °C / n.d.	5.6 °C	0.1 °C
PS87/106	81°13'N	141°10'E	-1.5 °C	-1.9°C	5 °C / n.d.	0.5 °C	-1.9 °C
ACEX	87°54'N	138°39'E	-1.5 °C	-1.9°C	6 °C / 12 °C	0.6 °C	-1.8 °C

Supplementary Table 4. Late Miocene monthly mean sea-surface temperatures and sea-ice concentrations obtained from model simulations[12, 13], using 450 ppm (values in red) and 278 ppm (values in blue) CO_2 scenarios as well as a pre-industrial scenario with 278 ppm CO_2 concentration (values in black). For location of the four sites see Fig. 1).

Location: ACEX: 87°54'N,138°39'E

CO2-level	Mio 450 PPM		Mio 278 PPM		PI 278 PPM	
	Ice conc	SST	Ice con,	SST	Ice conc	SST
Jan	0.55	-1.9	0.98	-1.9	0.98	-1.9
Feb	0.87	-1.9	0.98	-1.9	0.98	-1.9
Mar	0.96	-1.9	0.98	-1.9	0.98	-1.9
Apr	0.97	-1.9	0.98	-1.9	0.98	-1.9
May	0.93	-1.9	0.97	-1.9	0.97	-1.9
Jun	0.51	-1.7	0.91	-1.9	0.96	-1.9
Jul	0.01	-0.2	0.71	-1.9	0.90	-1.9
Aug	0	0.6	0.39	-1.8	0.86	-1.9
Sep	0	0.2	0.33	-1.8	0.89	-1.9
Oct	0	-0.6	0.68	-1.9	0.93	-1.9
Nov	0	-1.2	0.95	-1.9	0.96	-1.9
Dec	0.1	-1.7	0.98	-1.9	0.98	-1.9

Location: PS87/106: 81°13'N,141°10'E

CO2-level	Mio 450 PPM		Mio 278 PPM		PI 278 PPM	
	Ice conc	SST	Ice conc	SST	Ice conc	SST
Jan	0.49	-1.9	0.99	-1.9	0.98	-1.9
Feb	0.85	-1.9	0.99	-1.9	0.98	-1.9
Mar	0.95	-1.9	0.99	-1.9	0.98	-1.9
Apr	0.96	-1.9	0.99	-1.9	0.98	-1.9
May	0.93	-1.9	0.97	-1.9	0.97	-1.9
Jun	0.59	-1.8	0.92	-1.9	0.94	-1.9
Jul	0.04	-0.6	0.76	-1.9	0.87	-1.9
Aug	0	0.5	0.57	-1.9	0.84	-1.9
Sep	0	0.4	0.52	-1.9	0.86	-1.9
Oct	0	-0.3	0.77	-1.9	0.92	-1.9
Nov	0	-1.2	0.96	-1.9	0.96	-1.9
Dec	0.1	-1.7	0.98	-1.9	0.98	-1.9

Location: Site 910: 80°16'N,6°35'E

CO2-level	Mio 450 PPM		Mio 278 PPM		PI 278 PPM	
	Ice conc	SST	Ice conc	SST	Ice conc	SST
Jan	0.02	2.2	0.65	-1.4	0.92	-1.9
Feb	0.08	1.9	0.72	-1.6	0.91	-1.9
Mar	0.2	1.3	0.75	-1.6	0.93	-1.9
Apr	0.26	1.1	0.79	-1.8	0.96	-1.9
May	0.18	1.4	0.80	-1.8	0.94	-1.9
Jun	0.04	3.0	0.63	-1.6	0.92	-1.9
Jul	0	4.9	0.25	-0.8	0.82	-1.9
Aug	0	5.6	0.09	0.1	0.65	-1.9
Sep	0	5.0	0.05	0.1	0.60	-1.8
Oct	0	3.9	0.13	-0.4	0.69	-1.8
Nov	0	3.2	0.33	-0.8	0.85	-1.9
Dec	0	2.7	0.53	-1.2	0.90	-1.9

Location: Site 907: 69°15'N,12°42'W

CO2-level	Mio 450 PPM		Mio 278 PPM		PI 278 PPM	
	Ice conc	SST	Ice conc	SST	Ice conc	SST
Jan	0	8.0	0	4.6	0	2.4
Feb	0	7.9	0	4.4	0	2.4
Mar	0	7.9	0	4.4	0	2.6
Apr	0	7.9	0	4.4	0	2.7
May	0	8.3	0	4.9	0	3.3
Jun	0	9.8	0	6.2	0	5.1
Jul	0	11.9	0	8.0	0	7.0
Aug	0	12.6	0	8.7	0	7.5
Sep	0	11.4	0	7.8	0	6.4
Oct	0	9.8	0	6.3	0	4.8
Nov	0	8.7	0	5.2	0	3.3
Dec	0	8.2	0	4.8	0	2.6

Supplementary Table 5. Late Miocene alkenone-based (Uk'37) sea-surface temperatures (SST) at ODP Site 907. In some samples only trace amounts of alkenones were determined. In these samples, Uk'37 and SST values were not calculated. For age model see Supplementary Fig. 3. Data highlighted in red from ref. 7, all other from this study. All data are online available at http://dx.doi.org/10.1594/PANGAEA.855509

Depth (mbsf)	Age (Ma)	Sample number	C37:3 (area)	C37:2 (area)	UK'37	SST (°C)
80.2	5.13	9H5 40-42	7.36	3.78	0.339	8.95
81.2	5.23	9H5 140-142	5.24	2.66	0.337	8.88
83.2	5.42	9H7 40-42	12.10	7.51	0.383	10.27
84.2	5.52	10H1 88-90	Trace amounts	Trace amounts	--	--
85.2	5.62	10H2 40-42	5.72	2.19	0.277	7.06
86.2	5.71	10H2 140-142	Trace amounts	Trace amounts	--	--
87.2	5.81	10H3 90-92			0.278	7.10
88.0	5.88	10H4 17.5-19.5	10.59	6.27	0.372	9.94
89.2	6.00	10H4 140-142	1.78	1.33	0.427	11.61
90.4	6.10	10H5 110-112	7.66	7.37	0.490	13.53
91.7	6.21	10H6 87-89	21.52	8.44	0.282	7.20
92.3	6.26	10H7 1-3	13.59	5.92	0.303	7.86
92.9	6.37	11H1 11-13	28.50	5.95	0.173	3.90
93.5	6.46	11H1 73-75	135.28	17.72	0.116	2.18
94.4	6.54	11H2 11-13	61.97	36.90	0.373	9.98
95.5	6.65	11H2 121-123	75.54	43.90	0.368	9.81
96.4	6.73	11H3 60-62	21.41	17.80	0.454	12.42
97.0	6.85	11H3 117.5-119.5			0.443	12.10
97.4	6.95	11H4 12-14	22.11	16.42	0.426	11.58
98.0	7.07	11H4 69.5-71.5	191.56	122.42	0.390	10.48
98.5	7.18	11H4 121-123	12.04	12.21	0.504	13.92
99.0	7.28	11H5 18-20	18.20	19.41	0.516	14.31
99.6	7.40	11H5 77-79	3.19	4.97	0.609	17.12
100.0	7.50	11H5 122-124	2.50	3.13	0.556	15.51
100.5	7.60	11H6 20-22	5.73	7.89	0.579	16.22
101.1	7.72	11H6 77-79			0.550	15.30
101.4	7.79	11H6 111-113	trace	1.06		
101.9	7.89	11H7 7-9	1.97	2.88	0.593	16.65
102.5	8.02	12H1 20-22	8.11	6.66	0.451	12.33
103.1	8.14	12H1 77-79	5.32	7.57	0.587	16.47
103.6	8.25	12H1 127-129	3.62	4.74	0.567	15.86
104.0	8.34	12H2 22-24	30.42	29.31	0.491	13.54
104.5	8.44	12H2 68-70	Trace amounts	Trace amounts	--	--
105.1	8.56	12H2 127-129	1.35	1.88	0.582	16.30
105.5	8.66	12H3 20-22	Trace amounts	Trace amounts	--	--
106.1	8.78	12H3 77-79	Trace amounts	Trace amounts	--	--
106.6	8.88	12H3 127-129	5.39	5.54	0.507	14.02
107.6	9.09	12H4 77-79	2.10	2.62	0.555	15.48
107.9	9.17	12H4 111.5-113.5			0.545	15.20
108.5	9.29	12H5 19-21	1.97	2.78	0.585	16.40
109.1	9.42	12H5 82.5-84.5	1.43	2.12	0.597	16.76
109.5	9.51	12H5 123-125	1.91	4.44	0.699	19.85
109.9	9.59	12H6 12-14	Trace amounts	Trace amounts	--	--
110.5	9.71	12H6 69-71	Trace amounts	Trace amounts	--	--
111.1	9.84	12H6 130-132	4.38	8.43	0.658	18.61
111.5	9.92	12H7 16.5-18.5				
113.1	10.06	13H1 130-132	1.35	2.71	0.668	18.90
114.7	10.16	13H2 142-144	23.40	39.67	0.629	17.73
116.4	10.26	13H4 8-10	Trace amounts	Trace amounts	--	--
119.5	10.44	13H6 20-22			0.643	18.10
121.1	10.53	13H7 31.5-33.5	27.89	62.61	0.692	19.63
122.7	10.63	14H1 141.5-143.5	4.29	8.12	0.654	18.49
124.3	10.72	14H3 4-6	2.61	6.39	0.710	20.17
125.9	10.82	14H4 10-12	7.97	22.50	0.738	21.04
127.5	10.91	14H5 20-22	3.74	7.47	0.666	18.86
129.1	11.00	14H6 30-32	3.15	7.83	0.713	20.26
130.3	11.09	14H7 1-3	1.81	3.32	0.647	18.28
131.5	11.19	15H1 74-76	1.13	2.17	0.657	18.57
132.7	11.28	15H2 38-40	1.13	1.39	0.551	15.37
133.8	11.37	15H3 4-6	13.57	41.37	0.753	21.49
135.0	11.47	15H3 123-125	Trace amounts	Trace amounts	--	--
137.5	11.67	15H5 74-76	1.12	1.94	0.634	17.89
138.7	11.76	15H6 37-39	2.24	4.59	0.672	19.04
139.3	11.81	15H6 92-94	1.02	2.43	0.704	20.02
140.5	11.90	16H1 17-19	2.90	13.29	0.821	23.55
141.7	12.00	16H1 140-142	trace	1.07		
142.7	12.11	16H2 91.5-93.5	1.09	4.52	0.805	23.07
143.7	12.21	16H3 42.5-44.5			0.500	13.80
144.7	12.32	16H3 141-143	Trace amounts	Trace amounts	--	--
145.7	12.42	16H4 92-94	1.46	3.18	0.686	19.45
148.0	12.52	16H6 20-22	1.91	4.50	0.702	19.94
150.3	12.62	17H1 51.5-53.5	Trace amounts	Trace amounts	--	--
152.6	12.71	17H2 130-132	Trace amounts	Trace amounts	--	--
154.9	12.81	17H4 60-62	2.30	6.33	0.733	20.88
157.2	12.91	17H5 138-140	Trace amounts	Trace amounts	--	--
159.5	13.01	18H1 20-22	Trace amounts	Trace amounts	--	--
163.0	13.11	18H3 65-67	Trace amounts	Trace amounts	--	--
166.5	13.26	18H5 121-123	2.02	5.28	0.723	20.59
167.8	13.35	18H6 97-99	1.33	2.79	0.678	19.20
169.1	13.39	19H1 34-36			0.718	20.42
174.7	13.49	19H4 138-140	1.27	6.48	0.836	24.00

Supplementary Note 1

We thank the PS87 Geoscience Party for support in getting geophysical and geological shipboard data and sediments during the expedition.

The PS87 Geoscience Party (those not listed in the group of main authors) includes

Evgenia Bazhenova[1], Bernard Coakley[2], Anne de Vernal[3], Graeme Eagles[4], Hannes Eisermann[5], Tanja Fromm[4], Wolfram Geissler[4], Tanja Hörner[4], Haiyan Jin[6], Stefanie Kaboth[7], Bastian Kimmel[5], Henriette Kolling[4], Conrad Kopsch[8], Anne Kremer[4], Yngve Kristoffersen[9], Anna Kudryavtseva[1], Norbert Lensch[4], Seung-il Nam[10], Florian Petersen[11], Anna Katharina Prim[12], Florian Riefstahl[13], Albrecht Roloff[4], Isabel Sauermilch[13], Robert Spielhagen[14], Clara Stolle[4], Audun Tholfsen[9], Jessica Volz[13], Maria Winkler[4] & Mike Zwick[13]

[1]University of St. Petersburg, Universitetskaya 7-9, 199034 St. Petersburg, Russia.
[2]Department of Geology and Geophysics, University of Alaska Fairbanks, 903 Koyukuk Drive, Fairbanks, Alaska 99775-7320, USA.
[3]Département des Sciences de la Terre (GEOTOP), Université du Québec à Montreal, CP 8888 Montréal, Québec, H3C 3P8, Canada.
[4]Alfred Wegener Institute Helmholtz Centre for Polar und Marine Research (AWI), Am Alten Hafen 26, 27568 Bremerhaven, Germany.
[5]University of Hamburg, Mittelweg 177, 20148 Hamburg, Germany.
[6]Second Institute of Oceanography, State Oceanic Administration, 36 Baochubeilu, Hangzhou 310012, China.
[7]Department of Earth Science, University of Utrecht, Budapestlaan 4, Kamer O.332, 3584CD Utrecht, The Netherlands.
[8]ESYS GmbH, Schwedterstr. 34a, 10435 Berlin, Germany.
[9]Department of Earth Science, University of Bergen, Allégaten 41, 5007 Bergen, Norway.
[10]Arctic Research Centre, Korea Polar Research Institute (KOPRI), 26 Songdomirae-ro, Yeonsu-gu, 406-840 Incheon, Korea.
[11]Institute for Geosciences, University of Kiel, Otto-Hahn-Platz 1, 24118 Kiel, Germany.
[12]Institute for Geology and Paleontology, University of Münster, Corrensstr. 24, 48149 Münster, Germany.
[13]Department of Geosciences (FB5), Klagenfurter Str. 4, University of Bremen, 28359 Bremen, Germany.
[14] Helmholtz Centre for Ocean Research Kiel (GEOMAR), Wischhofstr. 1-3, 24148 Kiel, Germany.

Supplementary References

01. Stein, R. (ed.) *The Expedition PS87 of the Research Vessel Polarstern to the Arctic Ocean in 2014, Reports on Polar and Marine Research* Vol. 688 (Alfred Wegener Institute for Polar and Marine Research, Bremerhaven (2015).
02. Kaminski, M.A., Silye, L. & Kender, S. Miocene deep-water agglutinated foraminifera from IODP Hole M0002a, Lomonosov Ridge: Faunal constraints for the timing of the opening of the Fram Strait. *Micropaleontology* **55**, 117-135 (2009).
03. Kaminski, M. A., Silye, L. & Kender, S. Miocene deep-water agglutinated foraminifera from ODP Hole 909c : Implications for the paleoceanography of the Fram Strait Area, Greenland Sea. *Micropaleontology* **51**, 373-403 (2005).
04. Manum, S.B. *Decahedrella martinheadii* gen. et sp. nov. - a problematic palynomorph from the Northern Atlantic Miocene. *Palynology* **21**, 67-77 (1997).
05. Matthiessen, J., Brinkhuis, H., Poulsen, N. & Smelror, M. *Decahedrella martinheadii* Manum 1997 – a stratigraphicall and paleoenvironmentally useful Miocene acritarch of the high northern latitudes. *Micropaleontology* **55**, 171-186 (2009).
06. Schreck, M., Matthiessen, J. & Head, M.J. A magnetostratigraphic calibration of Middle Miocene through Pliocene dinoflagellate cyst and acritarch events in the Iceland Sea (Ocean Drilling Program Hole 907A). *Rev. Palaeobotany Palynology* **187**, 66-94 (2012).
07. Schreck, M., Méheust, M., Stein, R. & Matthiessen, J. Response of marine palynomorphs to Neogene climate cooling in the Iceland Sea (ODP Hole 907A). *Mar. Micropaleont.* **101**, 49-67 (2013).
08. Müller, P.J., Kirst, G., Ruhland, G., von Storch, I. & Rosell-Melé, A. Calibration of the alkenone paleotemperature index Uk37based on core-tops from the eastern South Atlantic and the global ocean (60 degrees N -60 degrees S). *Geochim. Cosmochim. Acta* **62**, 1757-1772 (1998).
09. Sikes, E.L., Volkman, J.K., Robertson, L.G. & Pichon, J.-J. Alkenones and alkenes in surface waters and sediments of the Southern Ocean: Implications for paleotemperature estimation in polar regions. *Geochim. Cosmochim. Acta* **61**, 1495-1505 (1997).
10. Prahl, F.G. & Wakeham, S.G. Calibration of unsaturation patterns in long-chain ketone compositions for palaeotemperature assessment. *Nature* **330**, 367-369 (1987).
11. Knies, J. *et al.* The emergence of modern sea ice cover in the Arctic Ocean. *Nat. Comm.* **5**, 1-5, doi: 10.1038/ncomms6608 (2014).
12. Knorr, G., Butzin, M., Micheels, A. & Lohmann, G. A warm Miocene climate at low atmospheric CO2 levels. *Geophys. Res. Lett.* **38**, L20701, doi: 10.1029/2011GL048873 (2011).
13. Knorr, G. & Lohmann, G. Climate warming during Antarctic ice sheet expension at the Middle Miocene transition. *Nat Geosci.* **7**, 376-381 (2014).
14. Channell, J.E.T., Amigo, A.E., Fronval, T., Rack, F., Lehman, B. Magneticstratigra-phy at Sites 907 and 985 in the Norwegian-Greenland Sea and a revision of the Site 907 composite section. In: Raymo, M.E., Jansen, E., Blum, P., Herbert, T.D. (Eds.), Proceedings of the Ocean Drilling Program, Scientific Results 162. Ocean Drilling Program, Texas A&M University, College Station, Texas, pp.131–148 (1999).
15. Backman, J., Moran, K., McInroy, D.B., Mayer, L.A., & the Expedition 302 Scientists. *Proceedings IODP 302* (Integrated Ocean Drilling Program Management International, College Station, Texas) (2006).
16. Frank, M. *et al.* Beryllium isotopes in central Arctic Ocean sediments over the past 12.3 million years: Stratigraphic and paleoclimatic implications. *Paleoceanography* **23**, PA1S02, doi:10.1029/2007PA001478 (2008).
17. Zachos, J.C., Dickens, G.R. & Zeebe, R.E. An early Cenozoic perspective on greenhouse warming and carbon-cycle dynamics. *Nature* **451**, 281-283 (2008).
18. O'Regan, M., Williams, C.J., Frey, K.E., & Jakobsson, M. A synthesis of the long-term paleoclimatic evolution of the Arctic. *Oceanography* **24**, 66–80, http://dx.doi.org/10.5670/oceanog.2011.57 (2011).

19. Sluijs, A., Schouten, S., Pagani, M., Woltering, M., Brinkhuis, H., Damsté, J.S.S., Dickens, G.R., Huber, M., Reichart, G.-J., Stein, R., Matthiessen, J., Lourens, L.J., Pedentchouk, N., Backman, J., Moran, K., the Expedition 302 Scientists, 2006. Subtropical Arctic Ocean temperatures during the Palaeocene/Eocene thermal maximum. *Nature* **441**, 610-613 (2006).
20. Sluijs, A. *et al.* Warm and wet conditions in the Arctic region during Eocene Thermal Maximum 2. *Nature Geosci.* **2**, 777-780 (2009).
21. Sangiorgi, F., *et al*. A 26 million year gap in the central Arctic record at the greenhouse-icehouse transition: Looking for clues. *Paleoceanography* **23**, PA1S04, doi:10.1029/2007PA001477, pp. 1-13 (2008).
22. Weller, P. & Stein, R. Paleogene biomarker records from the central Arctic Ocean (IODP Expedition 302): Organic-carbon sources, anoxia, and sea-surface temperature. *Paleoceanography* **23**, PA1S17, doi:10.1029/2007PA001472 (2008).
23. Stein, R. *et al.* Cenozoic Arctic Ocean Climate History: Some highlights from the IODP Arctic Coring Expedition (ACEX). *Developments in Marine Geology* **7**, 259-293 (2014).
24. Cabedo-Sanz, P. & Belt, S.T. Identification and characterisation of a novel mono-saturated highly branched isoprenoid (HBI) alkene in ancient Arctic sediments. *Org. Geochem.* **81**, 34-39 (2015).
25. Stein, R., Fahl, K. & Matthiessen, J. Late Pliocene/Pleistocene changes in Arctic sea-ice cover: Biomarker and dinoflagellate records from Fram Strait/Yermak Plateau (ODP Sites 911 and 912). *Geophysical Research Abstracts* **16**, EGU 2014-6895, EGU General Assembly 2014, Vienna, April 27 - May 02 (2014).
26. Belt, S.T. *et al.* A novel chemical fossil of palaeo sea ice: IP25. *Org. Geochem.* **38**, 16-27 (2007).
27. Ikari, M.J. & Kopf, A.J. Cohesive strength of clay-rich sediment. *Geophys. Res. Lett.* **38**, L16309, 5 pp., doi:10.1029/2011GL047918 (2011).
